# Using neural biomarkers to personalize dosing of vagus nerve stimulation

**DOI:** 10.1186/s42234-024-00147-4

**Published:** 2024-06-17

**Authors:** Antonin Berthon, Lorenz Wernisch, Myrta Stoukidi, Michael Thornton, Olivier Tessier-Lariviere, Pascal Fortier-Poisson, Jorin Mamen, Max Pinkney, Susannah Lee, Elvijs Sarkans, Luca Annecchino, Ben Appleton, Philip Garsed, Bret Patterson, Samuel Gonshaw, Matjaz Jakopec, Sudhakaran Shunmugam, Tristan Edwards, Aleksi Tukiainen, Joel Jennings, Guillaume Lajoie, Emil Hewage, Oliver Armitage

**Affiliations:** 1 BIOS Health Ltd., Cambridge, UK; 2 Université de Montréal and Mila-Quebec AI Institute, Montréal, Canada

**Keywords:** Vagus nerve stimulation, Neuromodulation, Biomarker, Heart failure

## Abstract

**Background:**

Vagus nerve stimulation (VNS) is an established therapy for treating a variety of chronic diseases, such as epilepsy, depression, obesity, and for stroke rehabilitation. However, lack of precision and side-effects have hindered its efficacy and extension to new conditions. Achieving a better understanding of the relationship between VNS parameters and neural and physiological responses is therefore necessary to enable the design of personalized dosing procedures and improve precision and efficacy of VNS therapies.

**Methods:**

We used biomarkers from recorded evoked fiber activity and short-term physiological responses (throat muscle, cardiac and respiratory activity) to understand the response to a wide range of VNS parameters in anaesthetised pigs. Using signal processing, Gaussian processes (GP) and parametric regression models we analyse the relationship between VNS parameters and neural and physiological responses.

**Results:**

Firstly, we illustrate how considering multiple stimulation parameters in VNS dosing can improve the efficacy and precision of VNS therapies. Secondly, we describe the relationship between different VNS parameters and the evoked fiber activity and show how spatially selective electrodes can be used to improve fiber recruitment. Thirdly, we provide a detailed exploration of the relationship between the activations of neural fiber types and different physiological effects. Finally, based on these results, we discuss how recordings of evoked fiber activity can help design VNS dosing procedures that optimize short-term physiological effects safely and efficiently.

**Conclusion:**

Understanding of evoked fiber activity during VNS provide powerful biomarkers that could improve the precision, safety and efficacy of VNS therapies.

**Supplementary Information:**

The online version contains supplementary material available at 10.1186/s42234-024-00147-4.

## Introduction

### Therapeutic potential and biological problem

The vagus nerve innervates most visceral organs in the thorax and abdomen, including the pharynx, larynx, heart, lungs, and gut (Johnson and Wilson 2018), as well as areas within the brain and central nervous system (Thompson et al. 2019). As such, it modulates functions such as respiration, circulation, and digestion. Abnormal vagus nerve activity has been associated with a variety of non-communicable diseases, including hypertension (Grassi 2009), heart failure (Kishi 2012), epilepsy (Ronkainen 2006), diabetes (Liao et al. 1995), cancer (Gidron et al. 2014), inflammatory diseases (Koopman et al. 2016), obesity (Kral et al. 2009) and eating disorders (Loper et al. 2021).

The vagus nerve’s diverse regulatory functions make it a prime target for therapies, most notably vagus nerve stimulation (VNS). VNS induces therapeutic effects by electrically stimulating the vagus nerve. By adjusting stimulation parameters, VNS can activate specific nerve fibers, resulting in different physiological effects. For example, stimulating large, myelinated A-fibers has an anti-epileptic effect (Bao et al. 2011); smaller, myelinated B- and possibly unmyelinated C-fibers are related to anti-inflammatory action (Sundman and Olofsson 2014), and B-fibers influence cardiac function (Qing et al. 2018).

VNS is FDA-approved to treat epilepsy, depression, obesity, and for stroke rehabilitation (U.S. Food and Drug Administration, PMAs P970003, P130019, P210007). It is being investigated as a therapy for heart failure (Zile et al. 2020), hypertension (Ntiloudi et al. 2019), inflammatory conditions (Pavlov and Tracey 2012; Kessler et al. 2012), traumatic brain injury (TBI) (Bansal et al. 2012), lung injury (dos Santos 2011; Reys et al. 2013) Alzheimer’s disease (Merrill et al. 2005), anxiety (George et al. 2008), chronic pain (Chakravarthy et al. 2015), tinnitus (Tyler et al. 2017), rheumatoid arthritis (Koopman et al. 2016), diabetes (Meyers et al. 2016) and obesity (Val-Laillet et al. 2010; Ikramuddin et al. 2014).

While cervical VNS has helped tens of thousands of patients with chronic disease (primarily refractory epilepsy), a persistent challenge is the therapy’s lack of precision with respect to target engagement. Response rate estimates range from 50 to 70% (Englot et al. 2015; Toffa et al. 2020; Batson et al. 2022) and side effects are commonly reported, including cough, voice alteration, laryngeal spasms, and local pain (Ben-Menachem 2001). Additionally, it can take up to 12 months of adjusting stimulation parameters before peak effectiveness is reached (LivaNova 2020).

This lack of precision is largely due to the complex morphology of the cervical vagus nerve (Fig. 2) and the fact that fibers associated with side effects tend to respond to stimulation more readily than those associated with desirable therapeutic effects (Fitchett et al. 2021). While fascicles are organized at least partially by the organs they innervate and the functions they mediate (Jayaprakash et al. 2022), the vagus nerve exhibits extensive branching and merging. Additionally, nerve morphology is unique for each patient and VNS parameter settings need to be adjusted accordingly. In regard to fiber engagement, the large myelinated fibers that innervate the mucosa and muscles of the neck, larynx and pharynx respond much more readily to stimulation (Gold et al. 2016) than most other fiber types (Yoo et al. 2013). This makes it difficult to avoid side effects such as cough, throat pain, dyspnea, and voice alteration (De Ferrari et al. 2017) when inducing a therapeutic response.

### Dosing nerve stimulation

In neurostimulation therapies, it is crucial to optimize stimulation parameters to achieve therapeutic effects and minimize side effects, i.e. to dose the VNS therapy. This is difficult due to the complex landscape of possible stimulation parameters that can be adjusted presenting a ’curse of dimensionality’ challenge, further exacerbated by inter-patient variability. As the number of parameters increase to achieve a more precise stimulation, the search space of possible parameter sets expands significantly. While optimal settings should be determined for each patient, brute force methods take too long to do safely or practically in the operating room or clinic, and would expose the patient to potentially dangerous side effects.

To address the lack of precision in target engagement of current VNS approaches, several studies have proposed selective VNS (sVNS) approaches including the selective blocking of undesirable nerve signal propagation (anodal block, kilohertz electrical stimulation block) and the application of novel electrode arrays and pulse shapes (Fitchett et al. 2021). While sVNS strategies have shown promise preclinically and in limited clinical use (Plachta et al. 2014; Aristovich et al. 2020; Dali et al. 2018; Pečlin et al. 2009; Ojeda et al. 2016), there are technological and scientific hurdles to overcome before these techniques are widely available in clinics. It is notable that all sVNS approaches extend the stimulation parameter space in order to describe selective stimulations, for example, by defining sets of complex pulse shapes or multiple electrode locations, increasing the parameter dimensions even further.

Faced with these problems, existing dosing methods tend to simplify the search space for users in one way or another. However, in doing so they reduce the possibility of finding an optimal and personalised dosing. For example, during dosing sessions, clinicians might choose from preset programs or adjust one parameter—typically current—until there are noticeable side effects (LivaNova 2020). Even within this highly restricted parameter space, it can take several months to reach effective stimulation settings for individual patients, as dosing adjustments are necessary to account for habituation.

### Objectives and outline of the present study

**Fig. 1:**
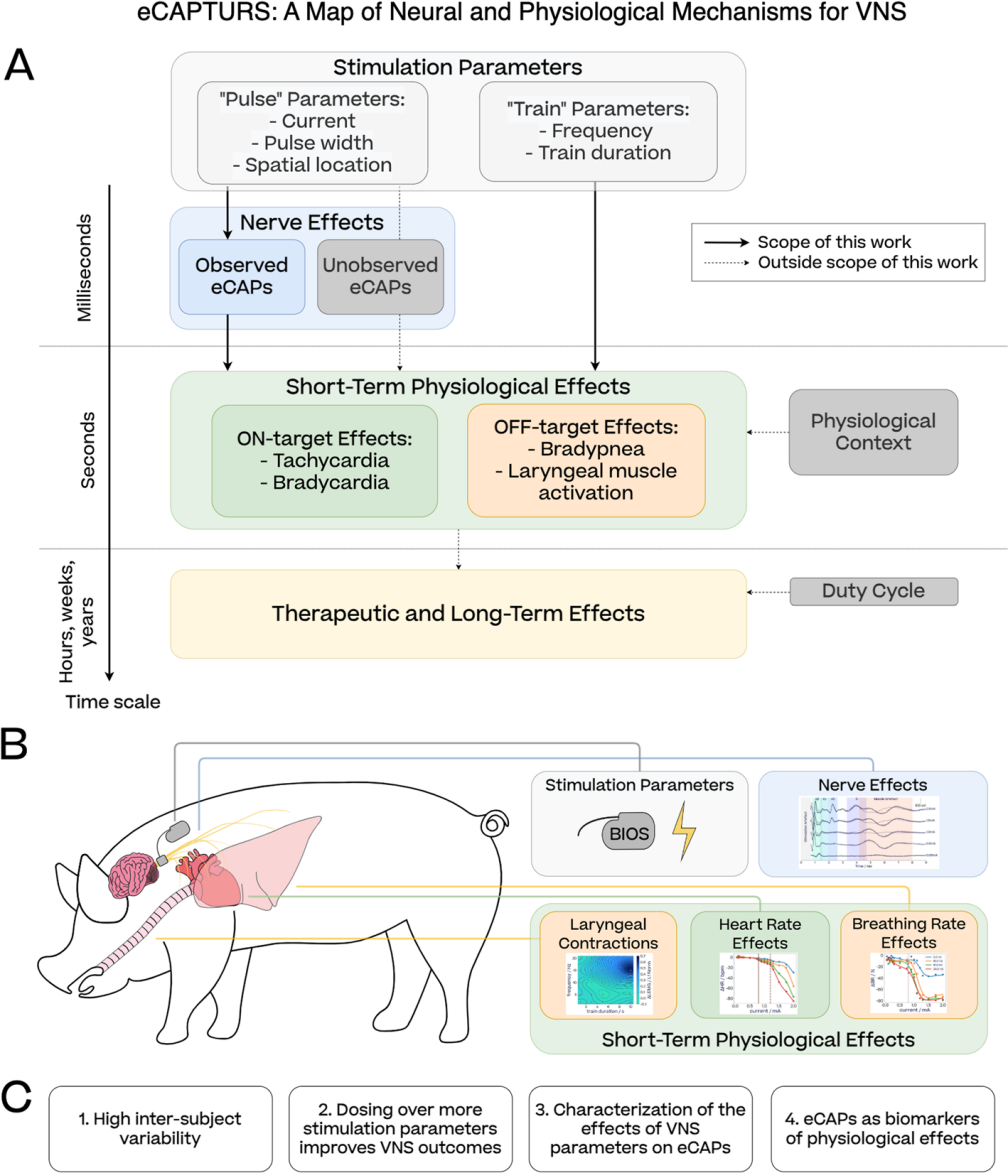
**A** Overview of the eCAPTURS framework (eCAPs To Unravel Responses to Stimulation), showing the relationship between stimulation parameters and responses on the nerve and the physiological level investigated in this study. The three solid arrows represent the main focus of this study. Light grey boxes indicate VNS parameters, as defined in North et al. (2022). The physiological state captures any bodily variables that might affect the short-term responses to stimulations, such as baseline heart rate or breathing rate, timing of the stimulation with respect to the cardiac (Ojeda et al. 2016) or breathing cycle (Sclocco et al. 2019) or anaesthesia (see Translation from anaesthetised to awake subjects section). **B** High-level representation of our VNS setting on porcine subjects: our custom neural interface delivers stimulations to the cervical vagus nerve while evoked fiber activity and short-term physiological effects (laryngeal contractions, heart rate and breathing rate changes) are recorded. **C** Key results reported in this study

Our main objective in this study is to address potential drawbacks of current clinical dosing strategies for vagus nerve stimulation (VNS) and explore novel approaches to identifying optimal dosing parameters. We achieve this by thoroughly examining the relationship between VNS parameters, the evoked compound action potentials (eCAPs), and physiological effects (Fig. 1A). This broader consideration of dosing aims to support the development of efficient and personalized dosing methods.

We carried out comprehensive studies employing a wide range of VNS parameters on anaesthetized swine (Fig. 1B), a suitable animal model for studying the human vagus nerve (Settell et al. 2020). In this context, “neural” specifically refers to eCAPs recorded on the cervical vagus nerve. We focus on VNS dosing for heart failure, where cardiac responses like tachycardia and bradycardia are considered “on-target effects,” while common side effects such as bradypnea or laryngeal muscle contractions are treated as “off-target effects” requiring mitigation. The methods presented in this study can be equally applied to other vagally mediated autonomic functions with similar off-target effects. We focus on short-term effects in the range of minutes. Evidence suggests that dosing of VNS parameters for physiological effects that arise within minutes, when applied periodically over much longer time spans, has the potential to improve clinically relevant outcomes (Dusi and De Ferrari 2021). Off-target effects of stimulation also typically appear within this time range.

Our analyses fall under four primary themes (Fig. 1C). First, we investigate the amount of inter-subject variability of physiological responses to VNS, which is often observed in current clinical applications of VNS, underscoring the need for personalized VNS dosing. Second, we evaluate how far exploration of a broad stimulation parameter space yields more desirable effects and fewer side effects compared to dosing protocols constrained to limited sets of parameters. Third, we explore how far stimulation parameters can be separated—for the benefit of an improved stimulation protocol—into two distinct groups. One subset, which we term *pulse parameters*, mostly determines the immediate neural response, the profile of neural fibers activated by a stimulation (Fig. 1A). Another subset, which we term *train parameters*, is responsible for integrating eCAPs into physiological effects. Each of these parameter groups suggests distinct optimization strategies. Fourth, we examine the potential of establishing a causal link between certain eCAPs and physiological target effects to enhance and expedite VNS dosing: once eCAPs are recognized as reliable markers for particular physiological responses, they can be optimized more quickly and with fewer side effects than the physiological targets themselves.

## Methods

### Surgical methods

The effects of VNS on physiological and neural response were examined in ten female swine (Yorkshire, (43.2±3.5)). Animal protocols were approved by the Institute of Animal Care and Use Committee of American Preclinical Services (Minneapolis, US). All animals were sedated with a mixture of Ketoprofen (2.0-3.5 mg kg^-1^), Tiletamine/Zolazepam (3.5-5.5 mg kg^-1^) and Xylazine (1.5-3.5 mg kg^-1^). Isoflurane (0-5%, ventilation) was used to induce anesthesia. Following intubation, the anesthesia was maintained with propofol (2-8 mg kg^-1^, i.v.). Mechanical ventilation was provided prior to VNS. During VNS, mechanical ventilation was turned off to measure the breathing response to stimulation. Normal body temperature was maintained between ${38}^{\circ }$C and ${39}^{\circ }$C using a heated blanket and monitored with a rectal probe thermometer. The depth of anesthesia was assessed by monitoring heart rate, blood pressure, respiration, and mandibular jaw tone.

Animals were positioned supine with both forelimbs and head extended to expose the ventral aspect of the neck. A 10 cm incision was made 2 cm right of the midline. Subcutaneous tissues were dissected and retracted to expose the underlying muscle layers. The sternomastoid muscle was bluntly separated and retracted laterally away from the surgical field. The carotid sheath was exposed and the vagus nerve was identified and isolated away from the carotid artery. The carotid artery was suspended with vessel loops and retained to the side of the surgical window with forceps.

**Fig. 2:**
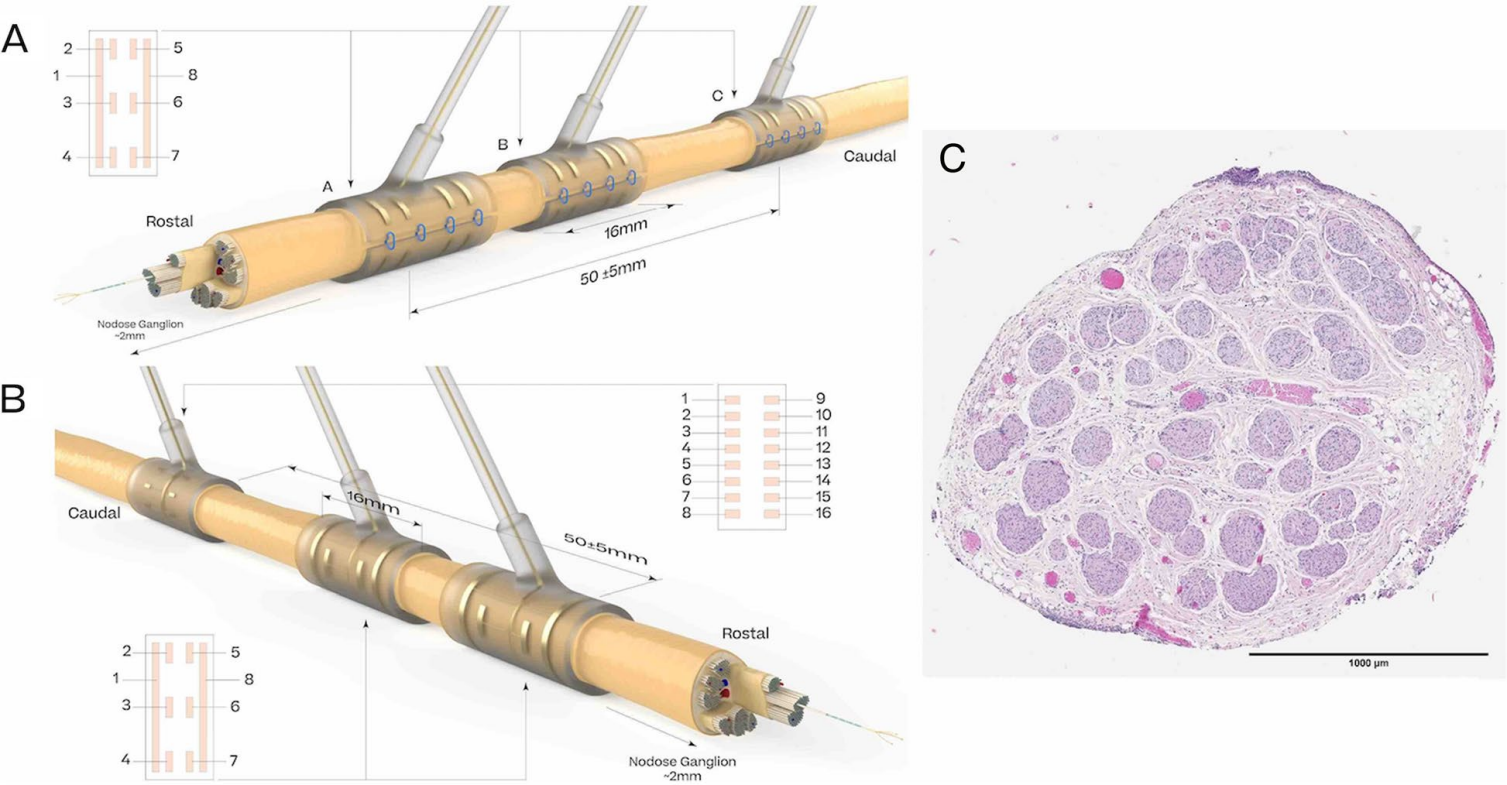
Representation of the cuff layout for Group 1 (**A**), Group 2 (**B**) subjects, and illustration of the histology of the vagus nerve (**C**)

Eight centimetres of the vagus nerve were stripped and isolated caudally of the nodose ganglion. Three cuffs (Microprobes, NC, US) were placed about 2 cm caudally to the nodose ganglion, with the most caudal cuff used for stimulation (Fig. 2). Recording was done from two cuffs (Cuff A and B), comprised of 2 ring-shaped contacts and 6 square contacts, circumferentially placed around the nerve. An inter-cuff distance of (50.0$\,\pm \,$5.0) was maintained between the centres of the stimulation cuff (Cuff C) and the most rostral recording cuff (Cuff A). Two cuff design variants were used for stimulation across the study cohort. Group 1 (subjects S1, S2, S3, S4, S6) used the same design as the recording cuffs. Group 2 (subject S5, S7, S8, S9, S10) used a cuff with 16 small square contacts in longitudinal pairs arranged in the same circumferential pattern around the vagus. An implantable EMG needle was inserted in the muscle left medial to the nerve for referencing the acquisition system to the tissue potential.

At the end of the study, the swine were humanely euthanised with an intravenous injection of Euthasol (390 mg mL^-1^). Death was confirmed by an absence of pulse on the ECG and arterial blood pressure signals.

### Custom neural interface

Cuff electrodes were connected to a custom neural interface utilising a 32-channel biopotential amplifier headstage (RHD2216, Intan Technologies) and a 16-channel bidirectional stimulator/amplifier headstage (RHS1116, Intan Technologies). The two headstages were controlled by an Artix 7 FPGA (Xlinix) sampling up to 30 kSamples/s per channel. Processes for data acquisition, data management, analysis and distribution were managed by Jetson TX-2 (Nvidia, CA, USA). This custom neural interface can be used on freely moving animals.

### Physiology monitoring

A two-lead electrocardiogram (ECG) was used in Lead I configuration using two subcutaneous probes. Laryngeal muscle activation were monitored by electromyography (EMG) using four surface patch electrodes arranged symmetrically in two bipolar derivations, similarly to Vespa et al. (2019). Briefly, one electrode pair was placed at 6 cm above and below the laryngeal prominence and one pair was placed horizontal at half the distance between the laryngeal prominence and the medial edge of the sternocleidomastoid muscle. Both ECG and laryngeal EMG signals were amplified and digitised using a bipolar recording headstage (RHD2216, Intan Tech). Breathing was measured using a SPR-524 ultra-miniature Nylon pressure catheter (ADI, Colorado, US) and arterial blood pressure was measured with a 2F catheter implanted in the right femoral artery. Breathing and blood pressure recordings were digitised at 1 kHz (PowerLab 16/35, ADI) and then visualised using LabChart (ADI, Colorado, US).

### Stimulation protocol

The vagus nerve was stimulated with regularly spaced rectangular pulses (see stimulation design in the Supplementary Materials). The following stimulation parameters were varied throughout this study: current (0.03-2.5 mA), frequency (1-50 Hz), pulse width (130-1000$\,\upmu$s), train duration (1-10 s), and spatial location. Stimulations were either monophasic (same polarity across all pulses) or biphasic with interphase delay (pulses have alternating polarity). Bipolar stimulation was delivered from different combinations of electrode pairs in the stimulation cuff. As a result, the depolarisation site where the eCAPs originate from (ie the current source) corresponds to either one or the other electrode, depending on the polarity. We use the following channel naming convention: two stimulation electrodes (e.g. A1-A8, for contact labels see Fig. 2 panel A, specifically the cuff schematic) and *cathodic pulse* (resp. *anodic pulse*) to indicate that the first (resp. second) electrode serves as the current source. Most stimulations performed used *longitudinal* pairs where the two contacts are located along the nerve (e.g. A1-A8), and a small subset of stimulations used *radial* pairs where the two contacts are located around the nerve (e.g. A2-A3).

Stimulation parameters were sequentially applied from a grid of parameter combinations. Prior to stimulation, the onsite electrophysiologist would mark when the hemodynamic indices had settled. We would apply the stimuli remotely, record the evoked neural and cardiovascular responses, and wait for the vitals to return to baseline before restarting the cycle. For stimulations affecting physiology, we would leave a minimum of 30 s between each stimulus. If the stimuli were deemed to be causing discomfort, we would not raise the charge further. If present, these would be in the form of heavy coughing or extended bradypnea or apnea. For this reason, it was not always possible to apply the same grid ranges to different subjects, when the focus was on physiological effect of VNS. This resulted in parameter sets that are only partially overlapping between subjects and made inter-subject comparisons less useful. With a focus on neural activation with short stimulation durations, however, similar sets of parameters were applied to different subjects and some direct inter-subject comparisons were possible.

### Data processing

#### Physiological effects

R-peaks in the ECG are identified by peak finding. The *heart rate* (HR) is calculated from RR distance and interpolated by the interpolate function (method pchip) from the Pandas (The pandas development team 2020) Python package. *Heart rate change* ($\Delta$HR) is defined by the difference of average heart rate before and shortly after the onset of stimulation. The three parameters (segment before onset of stimulation, gap time after onset, segment after gap) are chosen for each subject and parameter grid individually to optimize the model fit (measured by $R^2$) of a linear regression model with stimulation parameters as the explanatory variables. Examples of heart rate changes in response to stimulations are given in Supplementary Material.

Breathing pressure peaks are identified by peak finding, and the *breathing rate* (BR) is calculated by forward filling and expressed in breaths per minutes. The *breathing rate change* ($\Delta$BR) is calculated as the relative change (expressed in %) between the baseline breathing rate within 5 seconds before the stimulation onset and the minimum breathing rate between the onset of the stimulation and 3 seconds after the stimulation end. Examples of breathing rate changes in response to stimulations are given in Supplementary Material.

**Fig. 3:**
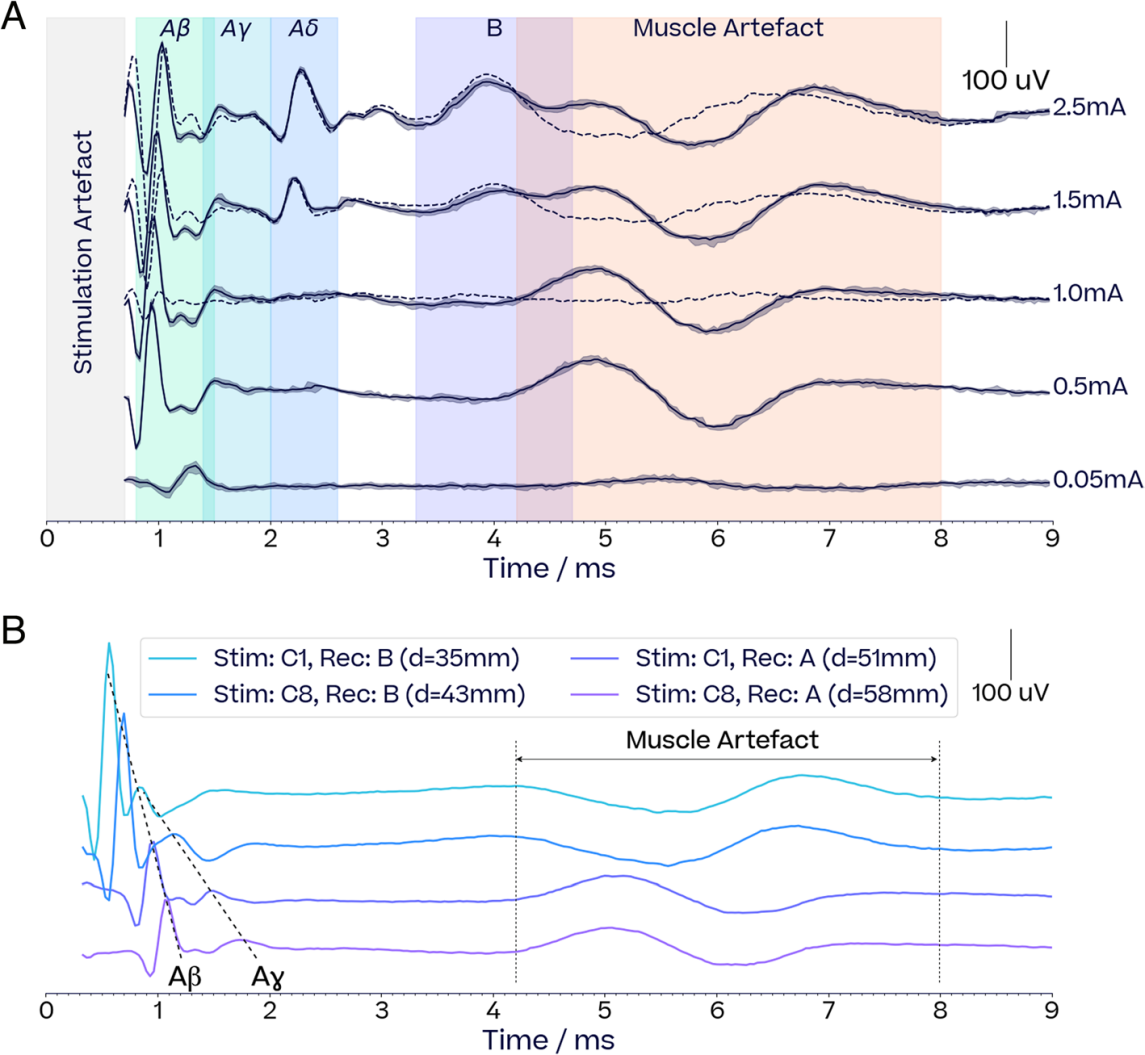
*Recordings of evoked compound action potentials (eCAPs)* **A** Neurograms at increasing currents (pulse width 500$\,\upmu$s, frequency 10 Hz, duration 1 s). Solid lines and confidence intervals respectively show the average and 5th/95th percentiles of the detrended response across the 10 pulses forming each stimulation train. For currents $>={1.0}$ mA, dotted lines show the average response after substracting the average 0.5 mA response to isolate the B-fiber eCAP from the muscle artefact. The stimulation artefacts are not shown as these are detrended responses. Raw recordings with the stimulation artefact are shown in the Supplementary Materials. **B** Propagation of the neural signal between different stimulation sites and recording locations for A$\beta$ and A$\gamma$ eCAPs (current 0.25 mA, pulse width 130$\,\upmu$s, frequency 10 Hz, train 1 s). Cuffs A, B and C correspond to Group 1 cuff layout, as illustrated Fig. 2. Distances between the stimulation and recording site are shown in the legend. The propagation of the A$\beta$ and A$\gamma$ eCAPs are consistent with known conduction velocities. The location of the muscle artefact between 4-8 ms is independent of the distance of each recording cuff to the stimulation cuff. The polarity changes since the bipolar electrode arrangement is reversed between the two cuffs

We observed two different effects on neck muscles: Activation of laryngeal muscles via the recurrent laryngeal branch by efferent A$\beta$-fibers as described in Nicolai et al. (2020) was observed in all subjects and validated for S6 via caudal vagotomy (Supplementary Material). In this work these are termed *laryngeal twitches*, and quantified by taking the $L_2$-norm of the EMG signal between 2.5 ms and 10 ms after the stimulation pulse. These laryngeal twitches also commonly appear in our neural recordings with similar responses than in EMG recordings (Supplementary Material). In this context, they are termed *muscle artefact* and discriminated from genuine neural activity from their lack of propagation as the distance between stimulation and recording locations vary (Fig. 3B). We also observed a phenomenon akin to a stronger contraction of the throat muscles, characterised by an increase in the EMG signal shortly after a stimulation. We term this effect *laryngeal spasm*, and propose to quantify it using the relative increase of the $L_1$-norm of the EMG signal one second before and after the stimulation train (Supplementary Material). A change of more than 0.3 (30% increase from baseline) is considered a strong spasm.

#### Neural signal

Evoked compound action potentials (eCAPs) from the vagus were isolated from the recordings after identifying and removing the stimulus pulses across a stimulation train. A 5th order Butterworth high-pass filter (200 Hz) was applied to correct for any exponential decays of the stimulation pulses. The signal was averaged across all stimulation pulses and eCAPs were classified into fiber types according to the Erlanger-Gasser classification (see Table 1) based on their conduction speed (see Fig. 3A). The authenticity of neural signals was validated by looking at their propagation between different depolarisation and recording locations. This is illustrated by Fig. 3B: as the relative distance between stimulation and recording sites increases, A$\beta$ and A$\gamma$ eCAPs shift in proportion to their respective conduction speed, while an EMG artefact appearing around 5-7 ms does not. We did not observe any C-fibers throughout our experiments. This is not surprising considering these are typically observed for currents well above our maximal current of 2.5 mA or with other specialist preparations (Tosato 2006; Yoo et al. 2013). We also did not observe any A$\alpha$-fibers, likely because their high conduction velocity (70-120 m/s) positions them within the stimulation artifact. However, throughout our experiments, the A$\beta$-fiber functionally resembled an A$\alpha$-fiber, consistently triggering laryngeal twitches. Therefore, for the purposes of this work, A$\beta$-fibers should be considered functionally analogous to A$\alpha$-fibers, similar to the findings reported in Nicolai et al. (2020).

**Table 1:** Conduction velocity, diameter, and functional classification of different fiber types (Gasser 1941; Manzano et al. 2008), along with proposed VNS physiological responses as considered in this work

Fiber type	Conduction velocity range (m/s)	Fiber diameter ($\upmu$m)	Functional classification	Proposed physiological response to VNS
A$\alpha$-fibers	70-120 m/s	12-22 $\upmu$m	Motor efferent	Innervate laryngeal muscles
A$\beta$-fibers	30-70 m/s	5-12 $\upmu$m	Sensory afferent	Activate laryngeal muscles
A$\gamma$-fibers	15-30 m/s	2-8 $\upmu$m	Motor efferent	Affects heart rate and breathing
A$\delta$-fibers	5-30 m/s	1-5 $\upmu$m	Sensory afferent	Affects heart rate and breathing, nociception
B-fibers	3-15 m/s	$<3\ \upmu$m	Parasympathetic efferent	Induce bradycardia
C-fibers	0.6-2.0 m/s	0.1-1.3 $\upmu$m	Visceral sensory afferent	Baroreceptor signaling to the brain

#### eCAP features

Activations from each fiber were computed by taking the $L_2$-norm of the linearly detrended signal within predefined time windows. The definition of these time windows are primarily derived from each fiber’s conduction speed, but also account for virtual cathode effects (Wikswo et al. 1991) for anodic pulses, which shift the depolarisation site of the afferent eCAPs in the rostral direction as current increases (Supplementary Material). Neural thresholds were defined as the current leading to 15% of the maximal activation recorded for each fiber, and computed by linearly interpolating dose-response curves. Potential contamination of B-fiber activation computation by muscle artefacts was removed by subtracting the response curve at 0.5 mA from responses to currents above 0.5 mA (see Fig. 3A).

### Data analysis

#### Train and pulse parameters

An important methodological choice we make in this study is separating VNS parameters into two groups–*pulse parameters* (applied current, pulse width, and electrode location) and *train parameters* (frequency and train duration). This is motivated by the observation that pulse parameters determine the evoked activation pattern while train parameters do not affect them at all, see Supplementary Material, Fig. S14. We note that the frequency range typical of clinical VNS do not lead to any refractory effect on the neural fibers. Therefore, the eCAP recruitment is governed by the local electrical fields generated by the current source and sink, which is in turn determined purely by the shape of each pulse. The limited effect of train parameters on vagal fiber activations has been observed in other works (Chang et al. 2020; Ahmed et al. 2021). In this study, we systematically explore the effects of these two sets of parameters and discuss how this might inform a new method for efficient VNS parameter optimization.

#### Alignment criterion for comparing neural activations with physiological effects in a subject

We consider the possibility of a causal connection between a neural and a physiological response to stimulation whenever the dose response curves (DRCs) agree in their points of onset and saturation (Verma et al. 2022; Blanz et al. 2023). We might term this approach the *DRC alignment criterion* for a potential causal connection between a specific fiber type activation and a specific physiological response. This is further discussed in Causal link between eCAPs and physiological effects section.

#### Gaussian process models

*Gaussian process models* (Rasmussen and Williams 2006) are used throughout the study for nonlinear regressions of response data. Nonlinear response curves and surfaces are obtained as mean functions of Gaussian processes with either squared exponential or Matern kernels. Hyperparameters are fitted by maximising the likelihood while imposing Gamma distributions as priors on parameters.

#### Regression functions

A *two-exponential curve* is computed by fitting a parametric curve of the sum of exponential functions: $f(x) =a_0+a_1 \exp (-x/b_1)+a_2 \exp (-x/b_2)$. The fit is obtained by minimizing the sum of residual squares over the five parameters. Similarly a *softplus* curve fits $f(x) = a_0 + a_1\log (1 + \exp ((x - a_3)/b$, and a *sigmoid* curve $f(x)=a_0 / (1 + \exp (-(x-a_1)/b)$. A *step-sigmoid* curve first fits a sigmoid curve to points with $x<c$ and then a second sigmoid curve to residuals with $x>c$.

#### Assessment of regression fit

To determine the fit of nonlinear regression models or the improvement of fit when comparing two regression models we use effective degrees of freedom of the kernel matrix and an *approximate F-test* (Hastie et al. (2009), Sec. 5.5.1).

## Results

Our experiments showed high inter-subject variability: the same set of stimulation parameters applied to different subjects led to different physiological responses (for examples see the supplementary material, Fig. S10 and Fig. S11). This variability can be attributed in part to differences in the physiological state of subjects, such as their baseline heart rates. However, it is inherent to the challenge of VNS dosing. Hence the results presented here demonstrate insights from subject-specific experiments.

The results are organised in three subsections: (1) the importance of a high-dimensional parameter search for individualized VNS dosing, (2) the effect of stimulation parameters on evoked fiber activity and (3) the link between evoked fiber activity and physiological responses.

### Response to vagus nerve stimulation using multiple parameters

Common clinical practice for VNS dosing reduces the stimulation parameter space to low- or one-dimensional searches. For example, the dosing procedure described in LivaNova (2020) mainly adjusts current amplitude, while total charge is commonly seen as the “dose” of a neuromodulation therapy (Zaaimi et al. 2008; North et al. 2022). However, such reduction of the parameter space fails to capture the complexity and range of evoked physiological responses (Supplementary Material, Section S4.2).

In this section we show that in many cases optimizing physiological responses to VNS over multiple parameters is preferable to optimization over a single parameter. We explore the advantage of considering multiple stimulation parameters or electrode contact locations when optimizing heart rate while controlling off-target effects such as bradypnea or laryngeal spasms.

#### VNS parameters for a trade-off between on-target and off-target responses

As on-target effects are often achieved by parameters that also evoke side effects, we explored a variety of systematic and extensive grids of VNS parameters for their effect on heart rate changes, which we consider an on-target effect, and breathing rate changes and laryngeal spasms, which we consider off-target effects.

**Fig. 4:**
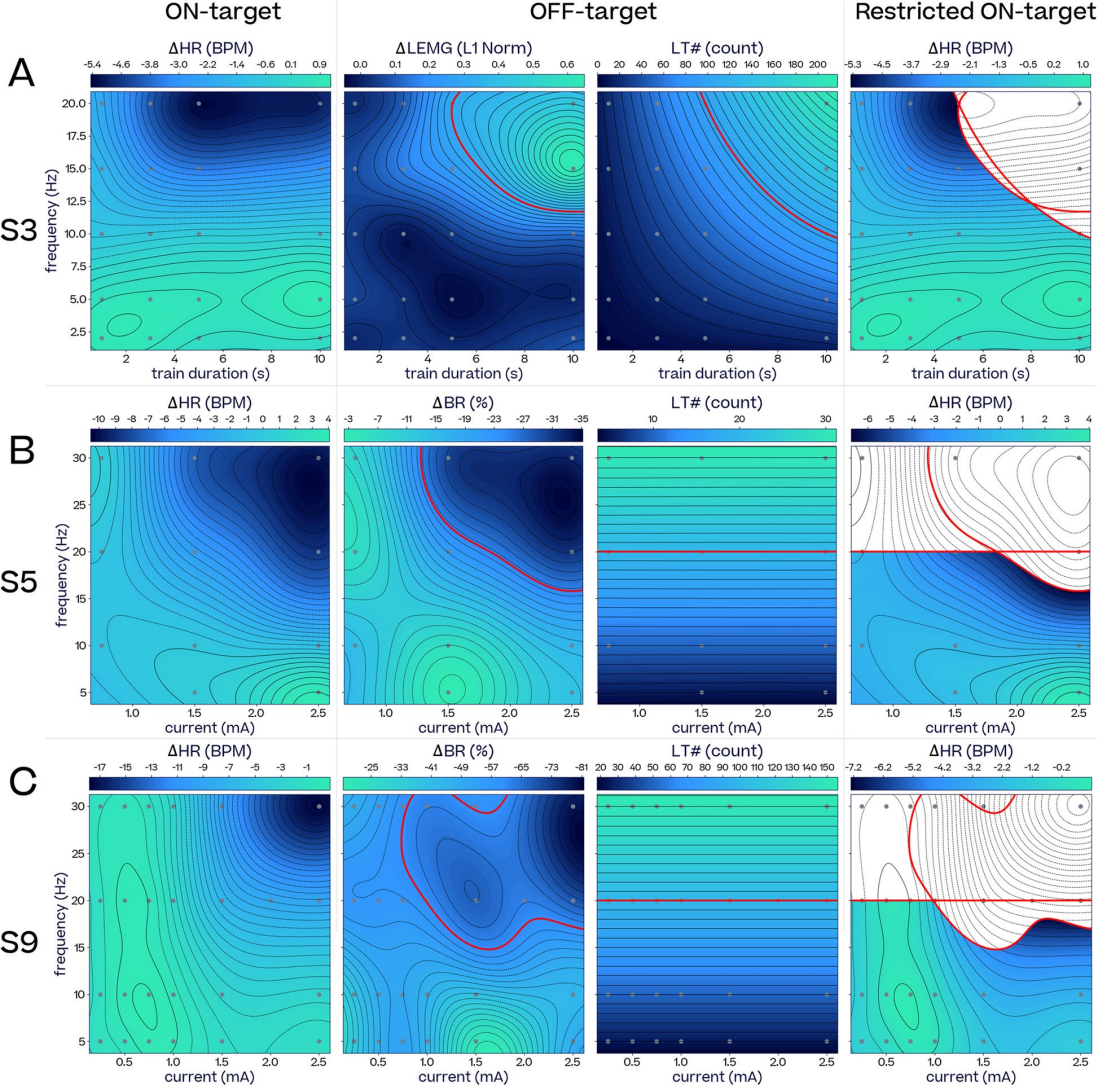
*On-target* $\Delta$*HR restricted by off-targets,* $\Delta$*BR,* $\Delta lEMG$ *and laryngeal twitch count (LT)*. **A** From left to right: Subject S3 $\Delta$HR, $\Delta lEMG$, LT and the frequency-train duration space available (non-blank) for $\Delta$HR adjustment when $\Delta lEMG > 0.3$ of maximum activation and $LT > 100$ should be avoided (current 1.5 mA, pulse width 250$\,\upmu$s) **B** From left to right: Subject S5 $\Delta$HR, $\Delta$BR, LT, and the frequency-current space available (non-blank) for $\Delta$HR adjustment when $\Delta BR < -25\%$ and $LT > 20$ should be avoided (train duration 1 s, pulse width 500$\,\upmu$s). **C** From left to right: Subject S9 $\Delta$HR, $\Delta$BR, LT and the frequency-current space available (non-blank) for $\Delta$HR adjustment when $\Delta BR < -60\%$ and $LT > 100$ should be avoided (train duration 5 s, pulse width 250$\,\upmu$s)

With three examples we illustrate how a search in a two dimensional parameter space (frequency combined with train duration or frequency combined with current) leads to improved optimization results for an on-target effect while avoiding two off-target effects, compared to a search along only one parameter. Changes in heart rate, laryngeal spasms and the number of laryngeal twitches (#LT) were recorded in subject S3 in response to frequency and train duration (Fig. 4A), while changes in heart rate, breathing rate and the number of laryngeal twitches were recorded in subjects S5 and S9 in response to frequency and current or train duration (Fig. 4B and C). Note that laryngeal twitches were evoked at all currents in these three experiments, so the number of laryngeal twitches off-target amounts to the number of pulses in the stimulation. The fourth column shows changes in heart rate when the off-target effect is kept under a given threshold. These off-target thresholds are chosen for illustration purposes, depending on each subject’s baseline physiological state (S3: $\Delta \text {LEMG}\le 0.3$ and $\#LT \le 100$, S5: $\Delta \text {BR}\ge -25\%$ and $\#LT \le 20$, S9: $\Delta \text {BR}\ge -60\%$ and $\#LT \le 100$). Additionally, a three dimensional parameter exploration is shown for Subject S6 in the Supplementary Materials (Fig. S12).

For Subject S3 (Fig. 4A), bradycardia with reduced laryngeal spasms and twitches is possible at 20 Hz if train duration is restricted to about 5 s. For Subject S5 (Fig. 4B), a bradycardia of -5 BPM can be reached while mitigating breathing rate changes and laryngeal twitches at currents of around 2-2.5mA and frequencies of 15-20Hz. For Subject S9 (Fig. 4C), a bradycardia of -5 BPM can be reached while mitigating breathing rate changes and laryngeal twitches with currents of around 2mA and frequencies of 15-20Hz.

#### Contact location affects the trade-off between on-target and off-target responses

**Fig. 5:**
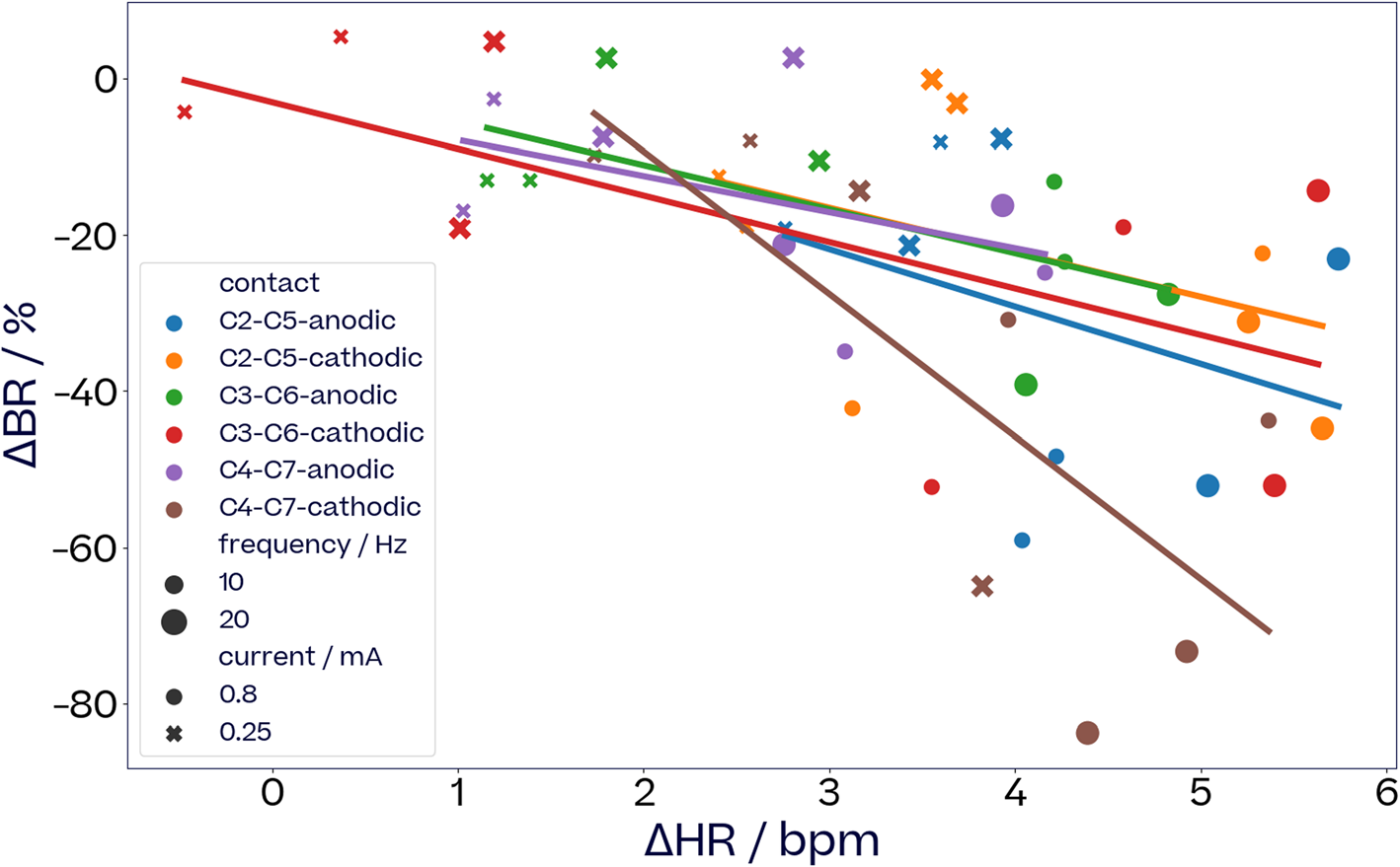
*Relationship of* $\Delta$*HR to* $\Delta$BR *dependent on electrode location*. Effect on HR and BR for six different stimulation locations (three electrode pairs, two polarities for each) pulse width was 500$\,\upmu$s with train durations of 3 s and 5 s, current is in mA and frequency in Hz. The train duration had no statistical effect on the relationship and is therefore not specifically labelled in the graphs. The overall location effect is significant (ANOVA, $F(5, 39)=3.6$, $p < 9.2\textrm{e}{-3}$). There is a significant difference in the slopes of C4-C7-cathodic compared to other contacts (ANOVA, $F(1, 43)=12.8$, $p < 8.9\textrm{e}{-4}$)

VNS recordings for subject S4 (Fig. 5) illustrate how contact location affects the trade-off between tachycardia (on-target) and strong bradypnea (off-target). Monophasic stimulations at 10 and 20 Hz, and 0.25 and 0.8 mA were applied with both polarities from three longitudinal contact pairs at different radial locations on the vagus (see Methods, Fig 2). Tachycardia and bradypnea were induced from all contact pair. However, for some contact pairs increase in heart rate came at the price of stronger bradypnea than for others. The overall location effect is significant (ANOVA, $F(5, 39)=3.6$, $p < 9.2\textrm{e}{-3}$). There is a significant difference in the slopes of C4-C7-cathodic compared to other contacts (ANOVA, $F(1, 43)=12.8$, $p < 8.9\textrm{e}{-4}$). This illustrates the potential of stimulating from well chosen spatial locations for optimizing a trade-off between on- and off-target responses.

### Effect of stimulation parameters on eCAPs

In this section we investigate the relationship between stimulation parameters and the evoked fiber activity. The first observation, which simplified the rest of the exploration, is that frequency and train duration (collectively called train parameters) have a minimal impact on the quantity and shape of the evoked compound action potentials within the frequency range of 2-50 Hz. This range includes frequencies used in approved VNS therapies (2-30 Hz). When current, pulse width, and electrode location remain fixed, neurograms are indistinguishable across different combinations of train parameters (MAPE: $0.05\%\pm 0.02\%$, Fig. S14, Supplementary Materials). This lack of temporal summation of evoked fiber activity across a stimulation train is also reported in Chang et al. (2020). This observation allows to separate the contribution of pulse and train parameters to neural and physiological responses to VNS, as depicted in Fig. 1.

#### Fiber recruitment depends on pulse parameters

**Fig. 6:**
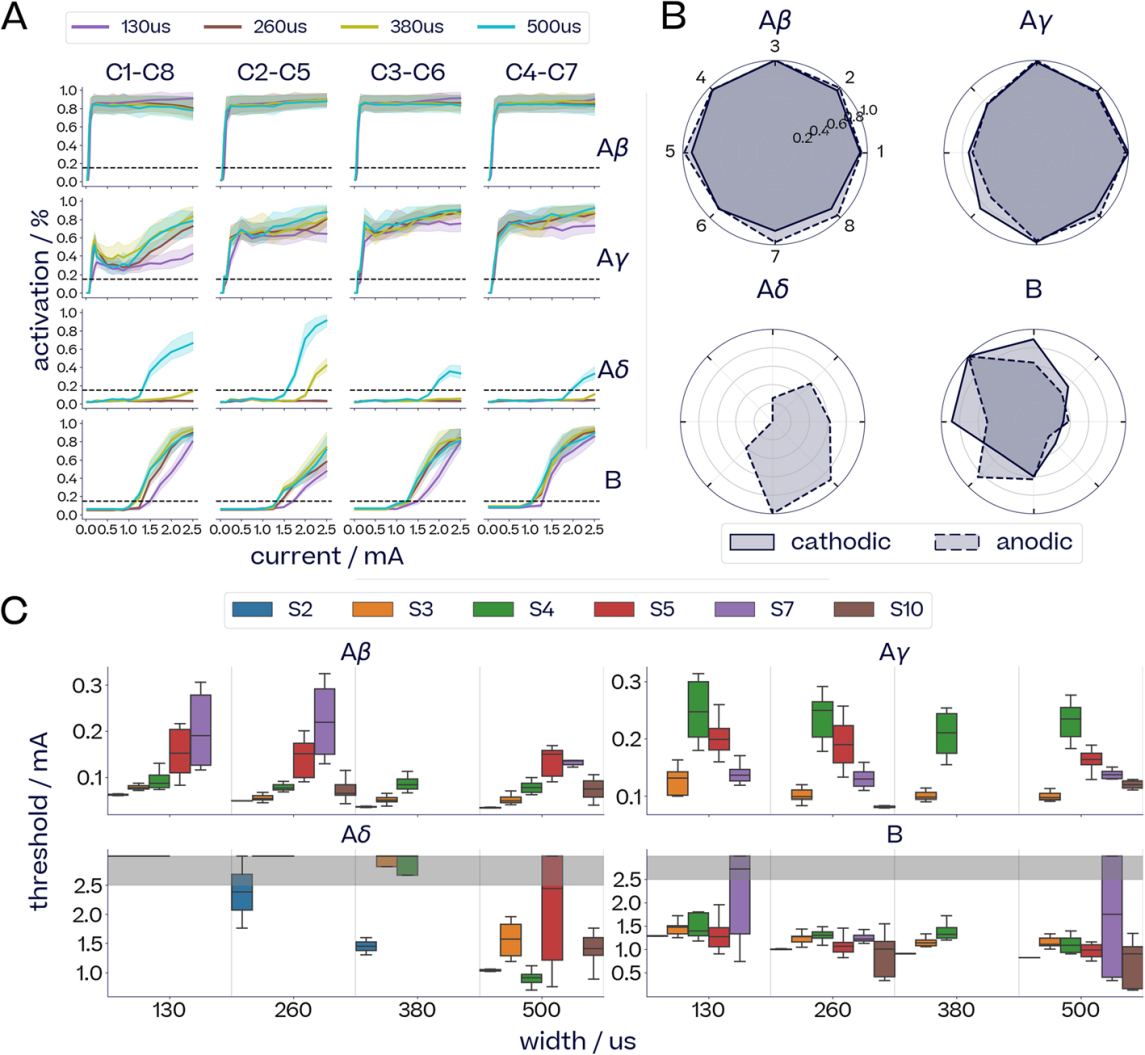
*Effect of pulse parameters on fiber recruitment*. **A** Dose response curves, normalised by the maximum found per fiber for subject S3. **B** Maximal fiber activation reached from each location in S5, normalised by the highest activation reached across locations for each polarity. **C** Distribution of neural thresholds across different subjects and pulse widths. The maximum amplitude was 2.5 mA, and points at 3.0 mA signal that the threshold was not reached by 2.5 mA. Pulse width was found statistically significant across all fiber types (ANOVA F-test: $p<5.7\textrm{e}{-17}$, $F=76.6$) and for each fiber type (A$\beta$: $p<8.5\textrm{e}{-3}$, $F=7.2$; A$\gamma$: $p<7.5\textrm{e}{-4}$, $F=12.1$; A$\delta$: $p<6.8\textrm{e}{-15}$, $F=94.5$; B: $p<1.4\textrm{e}{-3}$, $F=10.8$)

In contrast, variations in applied current, pulse width and electrode location (collectively called pulse parameters) lead to different fiber activation profiles. Current is the main driver of fiber recruitment. Fiber activation increases with current until it reaches saturation, giving rise to a sigmoidal dose-response curve (DRC). DRCs are characterised by the fiber activation (or neural) threshold and the saturation threshold, and these vary depending on the fiber type, pulse width and electrode location (see Fig. 6A for DRCs in a single subject with pulse widths from 130 to 500$\,\upmu$s, from four different stimulation locations).

Figure 6C shows the effect of pulse width on the distribution of neural thresholds for each fiber type across four subjects. For graphical display purposes, a nominal threshold of 3 mA was assigned when no activation was achieved over the current range to 2.5 mA. For all four subjects, pulse width has a statistically significant effect on neural threshold across all fibers (ANOVA F-test: $p<5.65\textrm{e}{-17}$, $F=76.6$). The effect of pulse width on the neural threshold is particularly pronounced in the case of A$\delta$-fibers.

Spatial selectivity for fiber activation was observed in subject S5, in which a higher density electrode cuff was used. Figure 6B shows the maximum activation reached for each fiber across eight longitudinal electrode pairs for current and width in the range 0.03-2.5 mA and 130-500$\,\upmu$s respectively. While the faster and larger A$\beta$-fibers are generally activated to the same extent regardless of stimulation location, smaller A$\gamma$-, A$\delta$- and B-fibers show distinctive spatially selective activation patterns. The full dose response curves for subject S5 across stimulation locations and pulse widths are shown in the Supplementary Materials (Fig. S16).

#### Inter- and intra-subect variability of nerve responses

We observe a wide distribution of neural thresholds across subjects (Fig. 6C). For each subject, the variation of the neural threshold of a given fiber across stimulation locations is represented by the spread of each box. The generally broader ranges of neural thresholds observed in Subject S5 and Subject S7 correspond to higher spatial selectivity owing to a different electrode layout.

#### Low-dimensional structure in neural responses across subjects

**Fig. 7:**
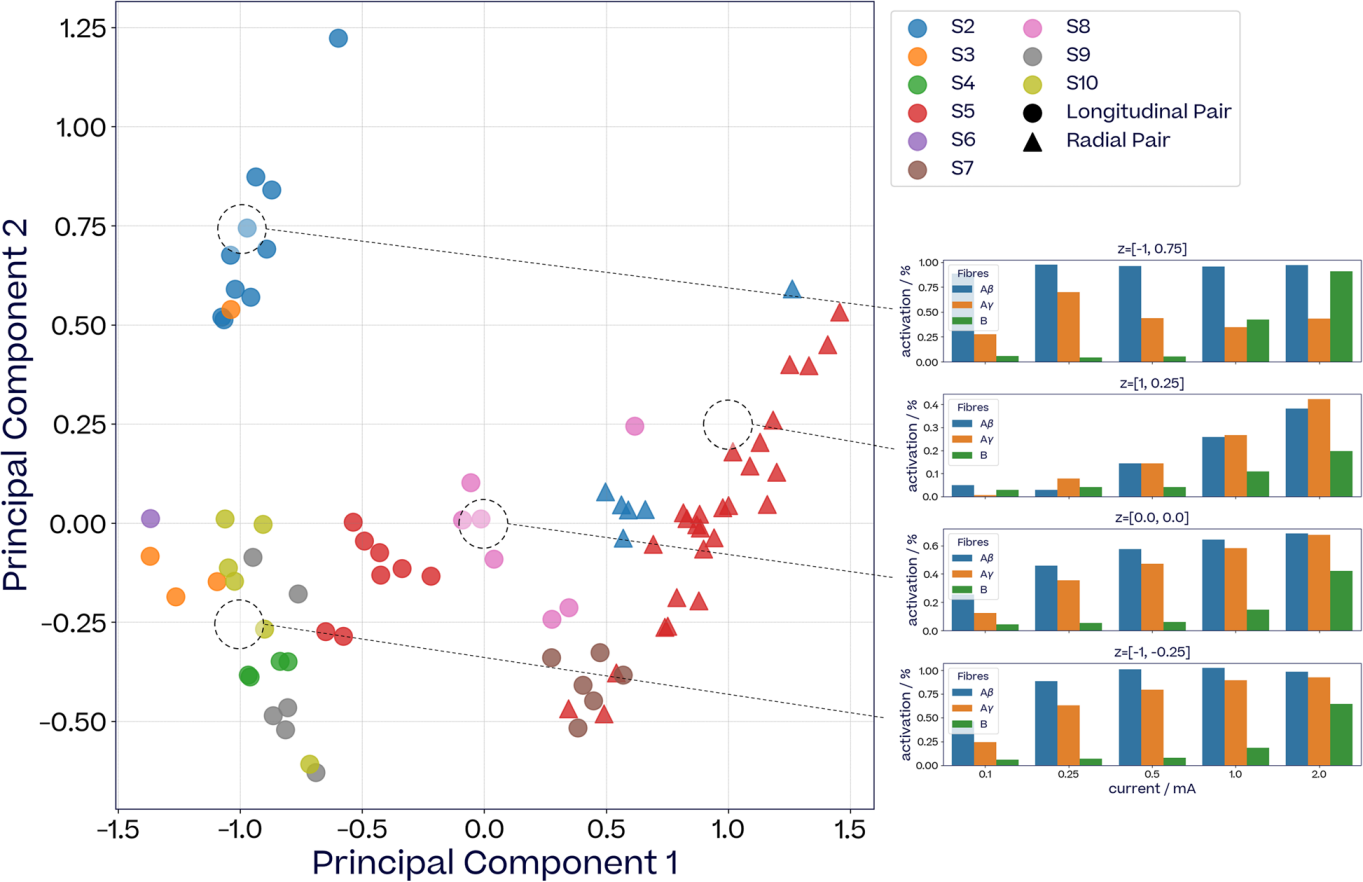
*2D PCA projection of evoked fiber responses across subjects and stimulation locations*. Each marker represents the 2D PCA projection of a 15-dimensions evoked fiber activations vector ((A$\beta$, A$\gamma$, B) $\times$ (0.1, 0.25, 0.5, 1.0, 2.0)mA, pulse width 260$\,\upmu$s) from a given stimulation location. Markers are color-coded by subject. Radial stimulation pairs are indicated with triangles. Bar plots on the right-hand side show the reverse projections of different points sampled from the two-dimensional space (labelled as *z*)

In order to visualise the underlying structure of the evoked fiber responses within and across subjects, we form 15-dimensions vectors from the activations of A$\beta$-, A$\gamma$- and B-fibers in response to five currents (0.1, 0.25, 0.5, 1.0 and 2.0mA) across 9 subjects. For each subject, we collect these 15-dimensions vectors for a pulse width of 260$\,\upmu$s across different electrode pairs, both longitudinal and radial. We then project these vectors into two dimensions using Principal Component Analysis (PCA). The two-dimensional space can be probed by sampling points in the 2D space and reversing the principal components projection in order to visualise the corresponding fiber activations in the 15-dimensions input space. The results are presented Fig. 7.

The resulting latent space present some interesting underlying structure that can be qualitatively interpreted. The first component seems to capture the readiness with which the fibers are recruited (left $\leftrightarrow$ low currents, right $\leftrightarrow$ high currents), while the second component might be representing the relative activation of A$\gamma$-fiber vs B-fibers (up $\leftrightarrow$ less A$\gamma$/more B, down $\leftrightarrow$ more A$\gamma$/less B). For example, the radial pairs are all located on the right-hand side of the space, indicating that they typically evoked fiber responses for higher currents than most longitudinal pairs.

The latent space also seems to capture some spatial information between the electrode pairs of each contact. For example, the longitudinal pairs of S5 present a loop-like structure (Supplementary Materials, Figure S17).

### Evoked compound action potentials as biomarkers of VNS responses

Physiological responses to VNS are influenced by the set of fibers activated at each stimulation pulse on the one hand, and by the train parameters such as frequency and train duration on the other hand. The functional anatomy of the vagus nerve is well known: large myelinated efferent A-fibers innervate the laryngeal muscle via the recurrent laryngeal nerve (Braund et al. 1988; Nicolai et al. 2020), myelinated afferent A-fibers such as A$\gamma$-fibers affect breathing and heart rate (Zaaimi et al. 2008), while B-fibers induce bradychardia (Yoo et al. 2016; Qing et al. 2018). A detailed description of different fiber types is summarised in Table 1. Nevertheless, empirical observation and validation of the relationship between fiber activity and physiological effects during VNS remains limited.

In this section, we examine the causal connection between the observed evoked compound action potentials (eCAPs) and physiological responses. Then, we illustrate how eCAPs provide a simplified mapping to physiological effects compared to stimulation parameters. Lastly, we show how train parameters contribute to the integration of eCAPs into different physiological effects.

#### Alignment of eCAPs and physiological effects

The alignment of neural and physiological response curves can be used to highlight causal connections between physiological responses and eCAPs (Verma et al. 2022; Blanz et al. 2023).

**Fig. 8:**
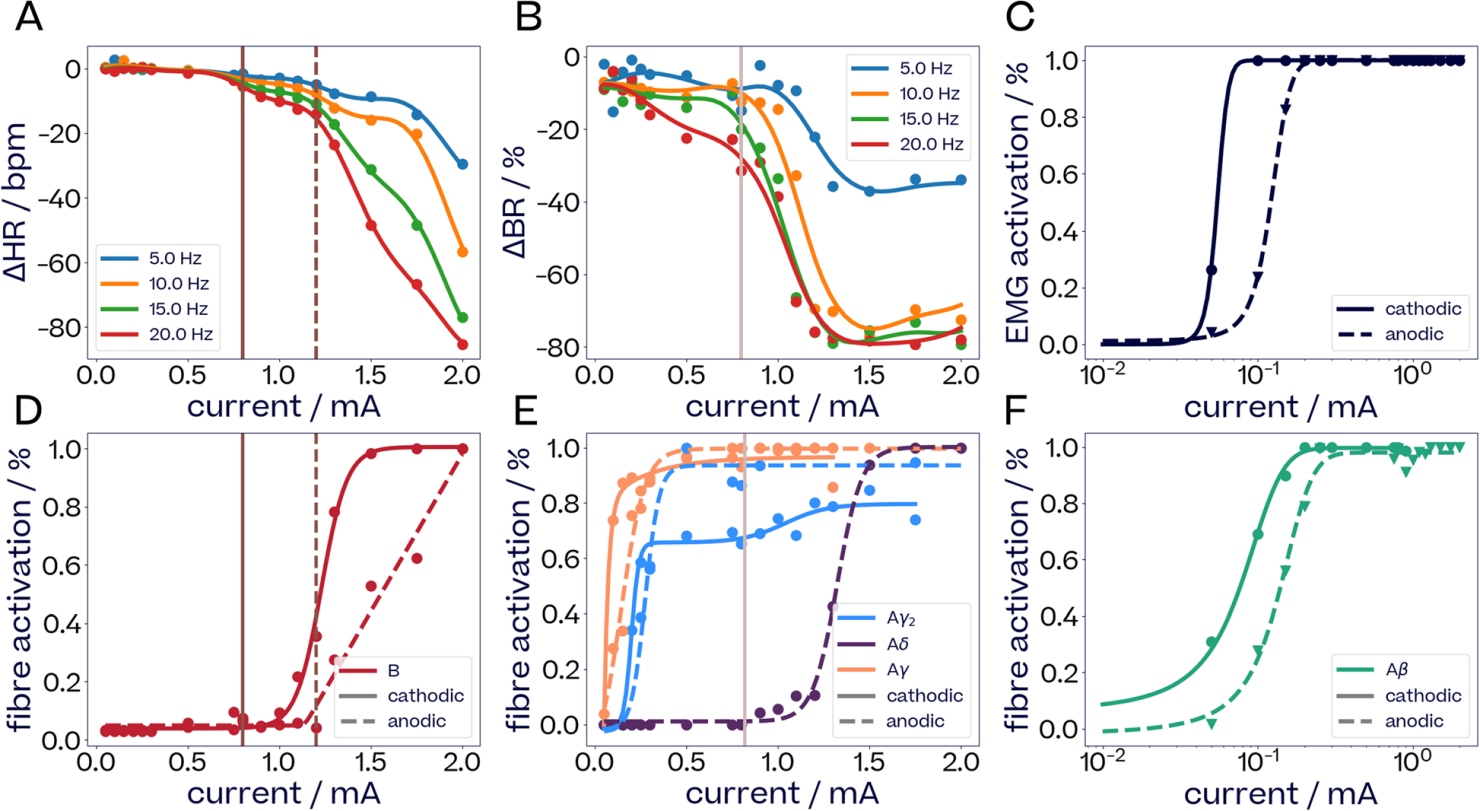
*Comparison of dose-response curves of HR and BR change, EMG activations and neural fiber activations for subject S6*. Left column (panels **A** and **D**): change in heart rate and the recruitment of B-fibers. Vertical lines (brown) for $\Delta$HR indicate the onset of mild bradycardia at 0.8 mA (solid) and of a stronger one at 1.2 mA (dashed). The same lines in the B-fiber activation plot correspond to onset of recruitment of cathodic B-fibers (solid) and anodic B-fibers (dashed). Middle column (panels **B** and **E**): change in breathing rate and the recruitment of A-fibers. A vertical line (beige) for $\Delta$BR indicates the onset of strong bradypnea at 0.8 mA. The same line in the A-fiber activation plot corresponds to onset of recruitment of anodic A$\delta$-fibers. Right column (panels **C** and **F**): EMG activation and the recruitment of A$\beta$-fibers. EMG activation happens at the same currents as A$\beta$-fiber recruitment for both cathodic and anodic pulses. General notes: frequency is not shown since it had no effect on eCAPs. Data points that fell below 0.9 of the maximum after a sequence reached its maximum have been removed as outliers. Trend lines for physiological and fibre activation observations are computed in order to make the general trends easier to discern. Methods used include sigmoid curves, sum of two sigmoids, two-exponentials, softplus as well as GP regression

Figure 8 compares changes in heart rate, breathing rate and EMG activation to the activation of A$\beta$-, A$\gamma$-, A$\gamma _2$- and B-fibers in subject S6. For increasing currents, we report $\Delta$HR and $\Delta$BR for frequencies between 5 Hz and 20 Hz, as well as EMG activation (panels A, B and C). Biphasic pulses with an interphase delay were applied, so that a single stimulation train consists of regularly spaced pulses with alternating polarity. The bottom row (panels D, E and F) shows the activations of the fibers putatively associated with each physiological effect. Since EMG twitches occur on a per-pulse basis, they are reported for both cathodic and anodic fiber activations (panels C and F), with a logarithmic scale x-axis to focus on low-current responses.

Mild bradycardia (Fig. 8A) is induced at 0.8 mA (solid brown vertical line), which aligns with the neural threshold of B-fibers for cathodic pulses (Fig. 8D). Note that at this current, anodic pulses do not trigger any B-fiber eCAPs. As such, B-fibers are activated by every other pulse of the stimulation train. Beyond 1.2 mA, anodic pulses begin to trigger B-fiber eCAPs as well, essentially doubling the frequency of B-fiber eCAPs per stimulation train (dotted brown vertical line). This aligns with stronger bradycardia above 1.2 mA. For higher currents, both anodic-triggered B-fiber activation and bradycardia continue to increase.

Mild bradypnea (Fig. 8B) is observed at low currents and seems to be related to A$\gamma$- and A$\gamma _2$-fiber activation (Fig. 8E). Strong bradypnea (Fig. 8B, beige line at 0.8 mA) aligns with an increase in A$\gamma _2$-fiber activation for cathodic pulses (Fig. 8E, beige line). As cathodic A$\gamma _2$-fiber activation reaches saturation around 1.2 mA, strong bradypnea levels off.

Laryngeal twitches (Fig. 8C) are evoked at low currents and closely follow A$\beta$-activations, for both cathodic and anodic pulses (Fig. 8F).

#### eCAPs simplify the mapping to physiology compared to stimulation parameters

**Fig. 9:**
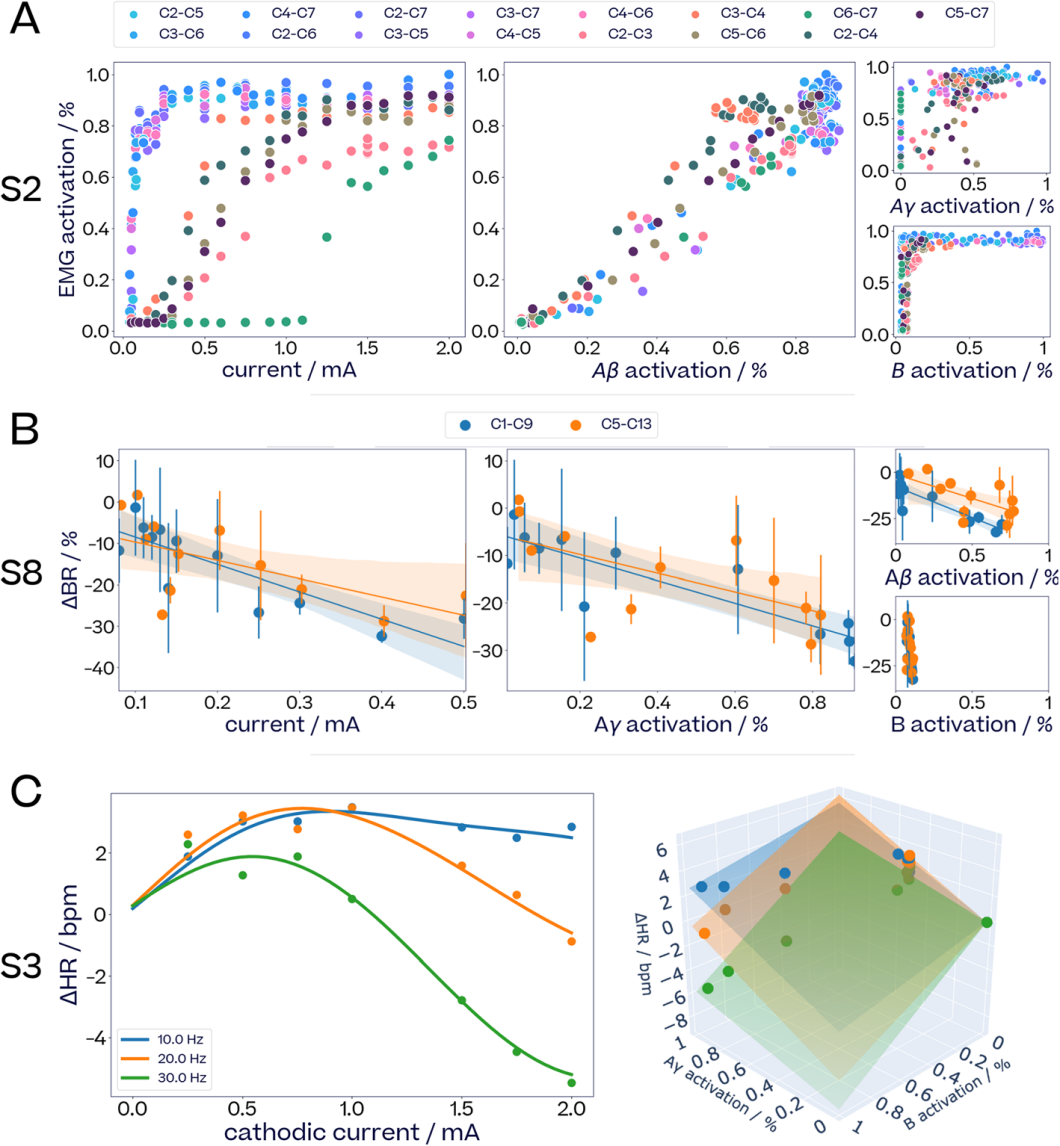
*Physiological effects, stimulation parameters and fiber activation*. **A** *Left*: Amplitude of VNS-induced laryngeal twitches in S2, plotted against current amplitude; *Middle and right*: activation of A$\beta$-fibers, and activation of A$\gamma$- and B-fibers across different radial and longitudinal stimulation locations. **B** Changes in breathing rate in subject S8 (pulse width 130$\,\upmu$s, frequency 5 Hz, train duration 1 s) in response to current (*Left*) A$\gamma$-fiber activation (*Middle*) and A$\beta$- and B-fibers activations (*Right*). Confidence intervals show one standard deviation for stimulations that were done more than once. Linear models are fitted for each of the two stimulation locations. The difference in slopes between the two contacts was significant for A$\beta$-activation (ANOVA, $F=7.68$, $p<0.01$), and not for current ($F=0.72$, $p=0.40$), A$\gamma$-fiber activation ($F=0.53$, $p=0.47$) or B-fiber activation ($F=0.29$, $p=0.60$). **C** *Left*: change in heart rate in response to current and frequency changes of stimuli in S3 (pulse width 500$\,\upmu$s, train duration 5 s). *Right*: the same change in heart rate, plotted in three dimensions against the activation of A$\gamma$- and B-fibers. 2D linear models are fitted for each frequency. (0, 0, 0) points are added to anchor the surfaces to the origin. All fiber activations are normalised based on the maximum fiber activations recorded across each subject

Since evoked fiber activity sits on the causal path between pulse parameters and physiological effects, eCAPs can provide a simplified mapping to physiological effects compared to stimulation parameters. Figure 9 illustrates this point for laryngeal twitches, breathing rate changes and heart rate changes.

Figure 9A shows the amplitude of laryngeal twitches with increasing current and A$\beta$-, A$\gamma$- and B-fiber activation across various longitudinal and radial electrode pairs in subject S2. The relationship between laryngeal twitches and pulse parameters is non-linear and varies greatly across electrode pairs. When plotting this effect against A$\beta$-fiber activation, however, the relationship becomes linear regardless of current amplitude and stimulation location. For A$\gamma$- and B-fiber activations, the relationship is once again non-linear.

Figure 9B shows breathing rate changes with increasing current and A$\gamma$-, A$\beta$- and B-fiber activation across two electrode pairs in Subject S8. Plotting breathing rate change over A$\gamma$-activations instead of current spreads out the stimulations under 0.2 mA, leading to a slightly better fit. Plotting over A$\beta$-activations results in significantly different slopes between the two contacts (ANOVA, $F=7.68$, $p<0.01$), whereas plotting over A$\gamma$-activations does not ($F=0.53$, $p=0.47$). The suggests that the activation of A$\beta$-fiber is not on the causal path of breathing rate changes, since otherwise there would be no any significant differences between the responses from different stimulation locations. B-fibers were not activated throughout the current range and therefore cannot explain the observed breathing effects.

Figure 9C shows heart rate changes in subject S3 for varying currents and frequencies. For frequencies 20 Hz and 30 Hz, low current amplitudes lead to tachycardia, before transitioning to bradycardia effect for higher currents, while 10 Hz stimulations only led to tachycardia even at high currents. When plotting these HR responses with respect to A$\gamma$- and B-fiber activations, the relationship becomes linear for each frequency. Notice the positive in contrast to the negative dependence of $\Delta$HR on A$\gamma$- and B-fiber activation, respectively, clearly shown in the two-dimensional linear mapping of activation to $\Delta$HR. Such linearisation of the HR effect via fiber activations was similarly observed for subject S4 (Fig. S18, Supplementary Materials).

#### Train parameters determine integration of eCAPs resulting in physiological effects

For a fixed set of pulse parameters, frequency and train duration determine how the neural activity evoked by each stimulation pulse integrates into physiological effects. In Fig. 8, when only cathodic pulses lead to B-fiber recruitment (brown line), HR decreases gradually as frequency increases from 5 to 20 Hz. When both cathodic and anodic pules lead to B-fiber recruitment (dotted brown line), there is a sharp increase in the rate of frequency-driven HR decrease. BR also decreases gradually with increasing frequency for currents above 0.5 mA, but seems to reach a plateau for frequencies above 10 Hz at high currents.

A striking effect of Fig. 9 is the difference in the integration of fiber activations that induced tachycardia from those that induced bradycardia. At currents below 1.0 mA, tachycardia is observed across all frequencies. Conversely, for currents above 1.0 mA, tachycardia is still observed at 10 Hz but transitions to bradycardia as frequency increases to 30 Hz.

## Discussion

In this section, we discuss the implications of the findings presented in this work for gaining a more comprehensive understanding of the mechanisms of action of VNS. We then present a novel VNS dosing procedure which leverages these insights to improve the efficacy and accuracy of VNS therapies. Finally, we address the limitations of this study.

Our results suggest two clear applications—the first practical, the second theoretical. The first application is how recordings of evoked fiber activity can be used to achieve personalized VNS dosing for research subjects or patients. Second, the results support the eCAPTURS framework (eCAPs To Unravel Responses to Stimulation) as depicted in Fig. 1A and discussed in Adaptability of eCAPTURS framework section. The figure serves as a guide for understanding the neural and physiological effects of VNS, while the version depicted in Fig. 1B reflects the experimental set-up of this paper. In the following we outline how it could be adapted for other VNS settings.

### Discussion of results

#### Variation between subjects

We observed considerable inter-subject variability and some intra-subject variability in physiological and neural responses to VNS (Fig. S10). At low charge dosages, most subjects exhibited tachycardia, but as charge increased, the tachycardia transitioned into bradycardia. Subject S6 (Fig. S10c), however, exhibited bradycardia in response to the whole range of VNS frequencies and currents. This was likely due to the high base heart rate (around 160 bpm) maintained throughout the experiment. Meanwhile, subject S4 only exhibited tachycardia for the range of charges that were deemed safe. Additionally, Fig. 4 illustrates how the most desirable VNS parameters vary greatly between subjects, highlighting the importance of personalized dosing in VNS therapies.

The variability in evoked fiber response can best be seen in Fig. 6C, which compares the neural responses of six subjects S2-5, S7, S10. For each subject (color), the spread of each box represents the variation of neural thresholds for different stimulation locations. Notice that not only did activation thresholds of fiber types differ between subjects, but also their variation over stimulation locations: some subjects showed very consistent activation thresholds independent of electrode contact, while for others thresholds differed considerably from contact to contact.

The relationship between fiber recruitment and physiological responses also showed inter-subject variability. Figure S15 compares the relationship between A$\gamma$- and B-fiber and heart rate between subject S3 and S4. While the relationship remains well approximated by 2D linear planes for each frequency, the slopes of these linear planes with respect to frequency vary between subjects.

The high inter-patient variability observed in physiological and neural responses to VNS as well as in the relationship between them suggests that personalized VNS dosing procedures are critical to the efficiency of VNS therapies.

#### Multiple parameters for optimal VNS dosing

One of the main challenges when using titration to optimize VNS parameters is that side effects can prevent one from reaching therapeutically effective regions of the parameter space. This is illustrated by examples in Fig. 4, where the region of the parameter space with desirable on-target effects often overlaps a region with undesirable off-target effects, an exclusion zone blanked out in white. However, if multiple parameters of a higher dimensional space are considered, a narrow region of satisfactory on-target effects avoiding unacceptable off-target effects can often be found just outside the boundary of the exclusion zone. If one simply titrated current or train duration along a fixed frequency such as 10 Hz, the best solution for maximum HR reduction with minimum side effects would be inferior to a solution found when one varies frequency in addition to current or train duration. Figure 4C, F, and I depict this for frequencies of 5, 15, and 20 Hz, respectively.

In the examples of Fig. 4 we only investigated on- and off-target optimization over two parameter dimensions (frequency-current, and frequency-train duration), which constitute low dimensional slices through a higher-dimensional parameter space. It stands to reason that searching over more parameters would improve the likelihood of finding satisfactory VNS parameters further.

Another dimension of VNS parameters is added through a choice of electrode locations as in Contact location affects the trade-off between on-target and off-target responses section. Figure 5 shows the relationship between $\Delta$HR, considered an on-target effect, and $\Delta$BR, considered an off-target effect, for six different contact pairs of a bipolar stimulation. This relationship changed with contact pairs, some showing a higher gain in $\Delta$HR for a smaller amount of $\Delta$BR than others. Our dosing method introduced in VNS dosing using neural recordings section could exploit such dependencies for an optimal trade-off between on- and off-target effects by choosing the right contacts in addition to optimizing other VNS parameters.

Overall, these examples show that considering as many dimensions of the parameter space in VNS as possible increases the opportunity to find a parameter set with a favourable balance between on- and off-target effects. This comes at a price, however: with every additional parameter the search space grows drastically and VNS dosing becomes increasingly difficult and time consuming. As we describe in VNS dosing using neural recordings section, we argue that measurements of evoked fiber activity allow to alleviate this issue and perform thorough searches of the parameter space quickly and safely.

#### Selective eCAP recruitment via pulse parameters

Selective VNS (sVNS) is a promising way to improve the precision of VNS therapies (Fitchett et al. 2021) by targeting some fibers or fiber types while avoiding the activation of others. The stimulations we applied in this study were restricted to square pulse shapes. Hence, fiber-selective stimulation depended on variation in pulse current, width or stimulation location (including polarity). Here we discuss the extend to which these stimulation parameters can achieve selective eCAP recruitment.

Throughout our experiments, pulse width had a significant effect on the evoked fiber activity: wider pulses were associated with lower activation thresholds for currents across all fibers (Fig. 6C). This follows from the strength-duration curve of activation thresholds relating pulse widths to currents (Irnich 2010). This effect enabled fiber-selective activation in the case of A$\delta$-fibers, which were almost never recruited for pulse widths below 260$\,\upmu$s but consistently activated above 500$\,\upmu$s. The effect of pulse width on A$\beta$-, A$\gamma$- and B-fiber activation was less pronounced. The activation threshold for fast A-fibers remained much lower than that for B-fibers regardless of pulse width.

Spatial sVNS exploits the phenomenon that different fibers can be activated from different contact locations. The refined electrode layout of the cuff design used in subject S5 demonstrates the potential for selective activation of either A$\delta$- or B-fibers (Fig. 6B) depending on contact location. Anodic stimulations from contacts 7 and 8 recruited A$\delta$-fibers and little to no B-fibers, while contacts 3, 4 and 5 recruited B-fibers and no A$\delta$-fibers for both polarities. Activation of A$\beta$- and A$\gamma$-fibers could only be reduced to around $80\%$ of their maximal activation be choosing specific spatial locations. A$\beta$- and A$\gamma$-fibers are the largest myelinated fiber types and as such they seem to be activated more uniformly across electrodes. Consequently, it might be difficult to suppress A$\beta$- and A$\gamma$-fiber activation while activating other fiber types based on current and electrode location alone.

These findings suggest that, although the simulation framework considered in this work allow for some selectivity over A$\delta$- and B-fibers, lower-threshold fibers such as A$\beta$- and A$\gamma$-fibers are not easily mitigated during high intensity stimulations. Using finer electrode layouts such as in Aristovich et al. (2020) might allow for more selective fiber recruitment. Additionally, further fiber selectivity might be possible by using more complex pulse shapes, as discussed in Limitations of the optimization in the eCAP space section.

#### Selective integration of eCAPs via train parameters

In selective VNS pulse parameters such as contact location or pulse shape are chosen in order to target specific fiber types within the nerve and consequently induce specific downstream physiological effects. We observed little if any influence of train parameters on evoked fiber activity in this study. Nevertheless, physiological selectivity of stimulations can be achieved via a judicious choice not only of pulse but also train parameters, an effect we term *selective integration* of eCAPs.

Prime examples of selective integration of eCAPs are seen in both cathodic and anodic stimulations of Fig. 9C. VN stimulations with the same pulse parameters and hence the same eCAP profile showed very different effects on HR depending on frequency, from causing tachy- to causing bradycardia. This drastic and qualitative change in physiological response can only be attributed to differential or selective integration effects of the very same eCAP pattern through a change in train parameters. We hypothesise that at low frequencies B-fiber do not exert their bradycardia effect, while faster A-fibers already exert their tachycardia effect. Meanwhile, increasing integration levels with higher frequencies lead B-fibers to exert their bradycardia effect, increasingly overpowering any underlying tachycardia effect. A similar effect has been found in mice in McAllen et al. (2018). Similarly, all stimulations in Fig. 4G, H induced the same evoked fiber activity, since only train parameters were changed throughout the grid. However, a low train duration and high frequency mitigates laryngeal spasms while maintaining a strong bradycardia effect.

Consequently, systematic exploitation of selective integration of eCAPs via train parameters provides an interesting avenue to targeting physiological effects in addition to selectivity at the eCAPs level via pulse parameters.

#### Alignment between recorded eCAPs and physiological effects

Although a formal causal analysis of the link between eCAPs and physiological effects is not applicable, as discussed further in Causal link between eCAPs and physiological effects section, alignment of neural with physiological DRCs might be suggestive of connections between specific fiber types and physiological effects such as bradycardia, tachycardia, bradypnea and laryngeal twitches.

Efferent B-fibers have previously been found to be the primary driver of cardioinhibitory effects (Qing et al. 2018), while tachycardia responses occur as a result of a decrease in neural parasympathetic drive caused by the activation of afferent sensory fibers of the VN Yamakawa et al. (2014); Ardell et al. (2017). This is in line with our experimental results. In S6, bradycardia effects closely follow the activation of B fibers in both cathodic and anodic pulses (Fig. 8). In S3 (Fig. 9C), tachycardia and bradycardia respectively follow the activation of A$\gamma$- and B-fibers, so that the overall heart rate responses can be explained with a linear regression model.

Previous studies concluded that bradypnea might be caused by A-fiber afferents from slowly adapting pulmonary receptors in the lungs Kubin et al. (2006), such as A$\gamma$-fibers. Our data suggest a similar mechanism. The bradypnea observed for subject S5 (Fig. 9B) seemed to be driven by train parameters (number of pulses) through a linear dependency. The slopes of these dependencies align most closely with the activation of A$\gamma$-fibers. For subject S6, mild bradypnea was observed at low currents, alongside the activation of A$\gamma$- and A$\gamma _2$-fibers (Fig. 8). A second increase in activation of cathodic A$\gamma _2$-fibers aligns with a second, stronger increase in bradypnea. Such a second raise after a first activation plateau might be attributed to the activation of an additional group of fascicles of the same type within the nerve. This effect is discussed further in Sec. 4.1.6.

Finally, laryngeal twitches are well known to be elicited by large myelinated efferent fibers via the recurrent laryngeal branch Nicolai et al. (2020). This is observed in our experiments in the right column of Figs. 8 and in 9A, where EMG activation follows closely the activation of A$\beta$-fibers.

#### Refined estimation of evoked fiber activity via multiple recording contacts

Throughout our experiments, activation of neural fibers were recorded from multiple locations. The amplitude of recorded eCAPs varied depending on the location and shape of the recording contacts. The variation is indicated with confidence bounds on the dose response curves. For example, Fig. 6A shows that activation of neural fibers could vary by $40\%$ across recording electrodes in subject S3.

We hypothesize that this variability provides valuable information about the underlying evoked fiber activity: eCAPs from a given fascicle will be larger from electrodes placed in their vicinity. The set of activation recorded from multiple local contacts therefore provides a sort of *activation signature* for every eCAP that can be used to characterise it further than via its propagation velocity. This is of importance since different fascicles within the vagus can share the same fiber type and propagation velocity but lead to different physiological effects. Isolating their respective eCAPs from the recordings based on their activation signature is therefore crucial to model the link between evoked fiber activity and physiological effects more faithfully.

#### Simplification of the relationship between VNS and physiological effects via eCAPs

As illustrated by Fig. 1, we suppose that pulse parameters only affect physiology via the evoked fiber activity. This would suggest that modelling physiological responses is more straightforward from fiber activity than from pulse parameters.

This is illustrated across various physiological effects in Fig. 9. The left hand size of the figure show laryngeal twitches and changes in breathing rate and heart rate with respect to various stimulation parameters. This mapping is complex: laryngeal twitches have a non-linear dependency on current, which varies with stimulation location; bradypnea is linearly related to the number of pulses, but the strength of this linear relationship depends in a non-linear fashion on current; the relationship between heart rate changes and current is non-linear and varies with frequency.

However, when plotting these physiological effects with respect to the appropriate fiber activity, this relationship is greatly simplified: laryngeal twitches depend linearly on A$\beta$-fiber activation; the slope of the relationship between breathing rate and the number of pulses is well explained by A$\gamma$-fiber activation; the tachycardia and bradycardia phases of heart rate changes respectively follow the activation of A$\gamma$- and B-fibers, and can therefore be captured across different frequencies by linear regression over the two dimensional (A$\gamma$, B) activation space.

The right-hand side panels of Fig. 9A and B illustrate the importance of using the appropriate fibers to explain a given physiological effect: the linear trend of laryngeal twitches breaks down when plotted against A$\gamma$- or B-fibers, and a similar effect occurs when plotting breathing rate changes against A$\beta$- and B-fibers.

### VNS dosing for clinical applications

#### VNS dosing using neural recordings

The high variability of physiological effects (Fig. S10, Supplementary Materials) as well as neural activations (Effect of stimulation parameters on eCAPs section) between subjects in the response to VNS suggests that personalized dosing is needed to optimize VNS settings for a desired therapeutic effect while avoiding side effects. Furthermore, the results in 3.1 suggest that existing dosing methods that adjust a single parameter, most often current, or cycle through preset programs are unlikely to lead to optimal settings. Stimulation parameters all influence (to a greater or lesser extent) the physiological responses of the subject. While personalized high-dimensional parameter search will likely lead to more precise physiological responses, the curse of dimensionality makes brute force search techniques impractical in the operating room or clinic.

We observe that neural recordings and the distinction between pulse and train parameters described in 3.2 and 3.3 and illustrated Fig. 1 can help tackle this challenge. Considering the pulse parameters separately from the train parameters while recording neural responses would simplify and clarify where variability is occurring in patients’ responses to VNS: the variability of the neural responses is captured by pulse parameters, while the variability of physiological responses is captured by train parameters and the evoked fiber activity. On the one hand, pulse parameters can be explored with the aim of optimizing for a preferred fiber activation profile, which is informed a priori by known organ functions of the different fiber types and can be refined during dosing based on alignments with physiological effects as demonstrated in Evoked compound action potentials as biomarkers of VNS responses section. For example, if the neural fulcrum introduced by Ardell et al. (2017) is a desired physiological target while trying to mitigate breathing effects, an optimization objective would be to activate B-fiber while minimising A$\gamma$-fiber activation. On the other hand, train parameters provide additional levers for controlling the depth of physiological effects and trade-offs between on- and off-target effects, as illustrated in Fig. 4.

With such a large parameter space an algorithmic approach to dosing would be critical to take full advantage of neural recordings in VNS dosing. While the pulse parameter space is high-dimensional, evoked fiber activity has an intrinsic timescale in the ms range and can be explored with limited physiological effects. This means neural responses can be optimized quickly and accurately across a large pulse parameter space. However, this would be highly challenging for manual approaches—as commonly used in current dosing procedures—which cannot take full advantage of the speed of neural responses and the high-dimensional pulse parameter space. Instead, an algorithmic approach to dosing would be able to consider high number of input parameters and efficiently search through multiple dimensions simultaneously. By handling the combination of neural and physiological responses to both pulse and train parameters, such an algorithm could guide the dosing procedure with optimal personalised stimulation parameter suggestions. In a companion paper (Wernisch et al. 2023) we describe, implement and test such an algorithmic approach to dosing, relying on Bayesian optimization, enabling an efficient, safe, and traceable optimization of neural and physiological responses to VNS.

#### Clinical relevance

Using neural recordings during VNS dosing offers the benefits of optimizing over a wide range of VNS doses in a short amount of time, improving responder rates and enabling therapies to be reoptimized over time.

Traditional dosing procedures rely on the observation of VNS-induced physiological effects to assess the effectiveness of a given set of stimulation parameters. Such physiological changes might happen in the order of a couple of seconds or minutes—e.g. in the case of short-term heart rate effects— but can take as long as hours for some autonomic function such as inflammatory responses which require blood samples to be collected. In contrast, neural activity is measured in a matter of milliseconds following a stimulation pulse, and the consistency of eCAPs across pulses of the same stimulation train as illustrated Fig. S13 suggests that evoked fiber activity can be assessed with as little as a single pulse. As a result, the neural responses to hundreds of potential stimulation parameters can be explored in the same amount of time required to assess the physiological effects of a single set of stimulation parameters.

In regard to responder rates, the organisation of fascicles within the vagus nerve varies greatly across patients, and is believed to be one of the main explanations for the high rates of non-responders to current VNS therapies. Approaches to tackle this include live imaging of the nerve in surgery via Electrical Impedance Tomography (EIT) (Ravagli et al. 2020) or ultrasound (Curcean et al. 2020) to determine the best location for the stimulation cuff. However, these approaches add to surgical complexity, and do not account for differences in the cuff-nerve interface. Instead, the fast exploration and optimization of the mapping between pulse parameters and evoked fiber responses allows one to capture the neural phenotype of each patient efficiently and safely.

Finally, using neural responses during VNS dosing would allow to reoptimize VNS therapies with less stress to the patient. While not used for VNS dosing today, Implantable Pulse Generators (IPGs) with neural recording capabilities or minimally invasive techniques, such as microneurography (Ottaviani et al. 2020; Verma et al. 2022), could enable neural recordings to be used periodically as efficacy decreases in response to electrode movement, scarring, or habituation.

#### Application to other VNS therapies

While this work focuses on VNS for heart failure and its effect on heart rate, the mechanisms presented here are relevant for dosing VNS therapies for other disorders, such as epilepsy and depression.

Although VNS has been successfully used to treat the latter two conditions for many years, side effects are common, and improvements in dosing procedures are needed. Side-effects reported by patients receiving these therapies, such as hoarseness, dyspnea or coughs (Ben-Menachem 2001; O’Reardon et al. 2006), are the same hindering the development of VNS therapies for heart failure. In both cases, varying stimulation parameters lead to trade-offs between the therapeutic efficacy and the severity of side-effects. For example, a review of past clinical trials for epilespy by Cramer et al. (2023) found that low frequency stimulations reduced the incidence or severity of side-effects, but to the expense of a decrease in efficacy. A recent extensive review of VNS for depression by Austelle et al. (2022) concluded that “there is still a lack of consensus on optimal stimulation parameters, dosing considerations and mechanisms”. This suggests that a more systematic and holistic approach to VNS dosing presented in this work has the potential to reduce side-effects and increase efficacy for these conditions.

### Limitations

#### Causal link between eCAPs and physiological effects

It is a basic assumption of VNS that eCAPs elicited by VNS lie on the causal path from stimulations to their physiological effects. However, eCAPs actually responsible for causing a specific physiological effect might elude recording. On the other hand, eCAPs recorded from a specific site on the VN might not be the ones involved in causing that physiological effect. Simple correlation, the co-occurrence of eCAPs with certain effects, is not necessarily causation.

Traditional causal inference based on statistical principles (Pearl 2009), which allows us to establish causal connections from observed data, is not applicable either. Such inference requires deliberate or gratuitous randomization on the process level. However, as we observed in this study, the relationship between stimulation parameters and eCAP patterns is essentially deterministic without any process noise.

Related methods for establishing a link between a fiber type activation and a physiological effect are based on correlation or regression analysis (Qing et al. 2018; Chang et al. 2020). Such methods typically depend on the correct choice of a regression model. For example, relying on correlation or a linear regression model for the typically highly nonlinear relationship between fiber activations and physiological responses can produce misleading results.

Therefore we used a weaker criterion to establish a possible causal link between certain eCAPs and a physiological effect: an alignment of DRCs of eCAP activations and response. The criterion requires that points of onset and saturation of the two DRCs agree. This criterion cannot prove a causal link but only suggest plausible candidates that might deserve further investigation. While similar approaches have been applied implicitly in the literature (for example in Verma et al. (2022) and Blanz et al. (2023)), here we have explicitly stated the principle and have applied it systematically in our data analysis, for example in Evoked compound action potentials as biomarkers of VNS responses section.

#### Limitations of recorded evoked fiber activity

In this study, we use a simple bound-based metric to measure the activation of individual neural fibers. However, this might give an incomplete view of the true evoked fiber activity. Firstly, cuff electrodes only measure the neural activity in their vicinity. Secondly, fascicles with similar conduction velocities can appear as a single eCAP in the neurograms. A fine understanding of the true evoked fiber activity is critical to predict and optimize downstream physiological effects. Computational modelling of the responses of neurograms from different radial locations could help uncover the geometry of the nerve and its evoked activity, and will be studied in future works.

#### Limited number of subjects

Given the high-dimensionality of the entire parameter space, as well as the time constraints imposed by surgery, broader exploration was preferred to consistency between subjects. We argue that if individual stimulation parameters have a significant importance in the dosing of a single subject, then they might be worth considering during the dosing of other subjects.

#### Translation from anaesthetised to awake subjects

The anesthetic agent used during VNS was propofol, which maintained a stable depth of anesthesia for recording neural and physiological activity. It is unclear whether anesthesia had an effect on the evoked fiber activity recorded on the vagus throughout our experiments. However, it certainly had an effect on downstream evoked fiber activity and physiological responses.

Rembado et al. (2021) showed that the effect of VNS on downstream evoked fiber activity in the central nervous system is modulated by brain states in nonhuman primates. This suggests anaesthesia might affect how vagal-evoked afferent activity is integrated and transmitted by the brain. Moreover, propofol is known to cause a fall in arterial pressure and a reduction in heart rate caused by central baroreflex sensitization (Whitwam et al. 2000). As a result, the integration of eCAPs into physiological responses might vary when translating to an awake setting.

Nevertheless, the invariance of neural responses across train parameters has been observed in other studies with different anesthesia profiles (Chang et al. 2020; Ahmed et al. 2021), indicating that the subject’s state should not affect the framework for VNS dosing presented in this work. Future work will further study the mechanisms between VNS parameters, eCAPs and physiological responses in the awake state.

#### Translation from porcine to human models

The approach taken by this study is non-destructive and could be used on humans to refine the understanding of the mechanisms of action of VNS in human subjects. While pig and human vagus nerves are very similar in diameter and composition (Pelot et al. 2020), making the porcine model the most suitable for clinical translation, morphological differences exist. The porcine vagus contains on average 10 times more fascicles than a human’s (Pelot et al. 2020). As a result, we hypothesise that understanding evoked fiber activity from neurograms and their relationship to physiological responses might be more challenging in porcine subjects than humans.

#### Limitations of the stimulation parameter space

The range of pulse widths and frequencies explored in this work covered ranges used in approved VNS therapies. For example, LivaNova (2020) uses pulse widths up to 500$\,\upmu$s and frequencies up to 30 Hz. Higher frequencies in the order of 100 Hz and 1kHz were not studied in this work, but have demonstrated promising properties for directionally-and fiber-selective stimulations (Patel and Butera 2015; Patel et al. 2017; Chang et al. 2022). The current was explored up to 2.5 mA due to hardware constraints. Neural dose response curves show most neural fibers were past or close to saturation at this current (e.g. Fig. 6A). However, this maximum ‘current was likely not enough to elicit any C-fiber responses, which have been observed for much higher currents or with specialist preparations (Tosato 2006; Yoo et al. 2013). The train duration explored in this study (1-10 s) were shorter than those typically used in current VNS therapies (e.g. 30 s in LivaNova (2020)). However, strong physiological effects were observed within this range of train durations without the need for longer durations that could potentially compromise the stability of the subject. We believe longer train durations as commonly used in current VNS therapies might not be necessary to achieve clinical effectiveness and might even be detrimental to the therapy by making side-effects more prevalent.

#### Limitations of the optimization in the eCAP space

In this work we only consider squared stimulation pulses. As studied Effect of stimulation parameters on eCAPs section, this means that the evoked fiber activity only depends on current amplitude, pulse width and stimulation location. However, this might limit the extent to which specific fibers can be activated while mitigating others: while pulse width and stimulation location can be optimized to preferentially target B-fibers, further selectivity might be required to fully mitigate other fibers with significantly lower neural thresholds such as fast A-fibers. A possible solution could be to use non-squared pulse shapes, which have demonstrated promising results towards selective fiber activation (Vuckovic et al. 2008; Pečlin and Rozman 2014; Dali et al. 2018, 2019). Although more complex pulse shapes would bring additional pulse parameters to consider, the speed and safety advantages of optimizing within the eCAP space would allow to deal with this increase in dimensionality of the input space.

#### Adaptability of eCAPTURS framework

The key results of this study informed the design of the eCAPTURS framework depicted in Fig. 1A. This framework can guide one’s understanding of neural and physiological mechanisms of VNS. While the version of this framework depicted in Fig. 1 is reflective of the specific experimental set-up described in Methods section, it can easily be adapted for experimental set-ups that utilize different VNS settings.

One example of how the framework could be adapted is by incorporating new pulse parameters from complex waveforms such as a depolarizing pre-pulse as studied by Vuckovic et al. (2008) or chopped pulses as studied by Qing et al. (2015) and Dali et al. (2019). The latter case is a particularly interesting use of the framework as the intra-burst frequency parameter used in chopped pulses would be considered a pulse parameter as it affects evoked fiber activity by leveraging the refractory periods of different fiber types, while inter-burst frequency would remain a train parameter as it does not significantly affect evoked fiber activity.

Another example of how the framework could be adapted is the inclusion of a closed-loop control mechanism (Bilgutay et al. 1968; Tosato 2006; Ugalde et al. 2014; Ojeda et al. 2016; Mylavarapu et al. 2023). These studies use live monitoring of the RR interval as an input for determining the timing and/or parameters for stimulating the nerve. In this case, specific delays from R peaks in the ECG as seen in studies by Ojeda et al. (2016) and Ugalde et al. (2014), could be included in the framework as “train parameters” as timing of the stimulation impacts physiological effects but is not expected to influence evoked fiber activity. In this example, an arrow would likely connect the “Physiological Context” box and the “Train Parameter” box depicted in Fig. 1A.

In addition to adding new stimulation parameters, studying VNS for other chronic conditions would require adding or removing physiological effects. The physiological effects listed in Fig. 1 are those that are most relevant to VNS for HF. Other physiological effects of VNS that might be commonly considered include dysphonia (Ardesch et al. 2010), biomarkers of inflammation (Kwan et al. 2016), and muscle activity in the gut (Lu et al. 2018). As we continue to deepen and broaden our experimental work, this framework will be extended to capture a more complete picture of the relationship between VNS, neural effects, and physiological changes.

## Conclusion

Choosing the right stimulation parameters in VNS has been one of the primary obstacles impeding its wider application to treat various chronic diseases. This study explores the relationship between VNS parameters, evoked fiber activity and short-term physiological responses to stimulation by exploring a wide range of systematic sets of VNS parameters. First, we observe high inter-patient variability, and find that increasing the range of stimulation parameters considered in VNS dosing improves outcomes; however, this comes with the cost of more extensive and time-consuming procedures, which are impractical under clinical constraints. Next, we examine the effect of stimulation parameters on evoked fiber activity. We differentiate between pulse parameters and train parameters, which makes this exploration faster and safer than for physiological responses. Lastly, we demonstrate how evoked fiber activity simplifies the mapping to physiological responses compared to stimulation parameters. Together, these findings suggest that dosing strategies harnessing insights from evoked fiber activity could explore and optimize over the stimulation parameter space more thoroughly and in a shorter amount of time than existing dosing procedures. This would lead to better therapeutic outcomes in fewer clinic visits for the patient and enable a new kind of precision medicine with personalized, safe and efficient VNS dosing. Future works will attempt to demonstrate and validate such calibration procedure in a pre-clinical setting.

### Supplementary Information

Supplementary Material 1.

## Data Availability

The data supporting the findings of this study is openly available in downloadable format on the oSPARC platform (https://discover.pennsieve.io/datasets/349). A simulation framework for optimal personalized dosing as described in Section 4.2.1 is available for demonstration purposes on the same platform (https://osparc.io/study/861f8d70-a2d4-11ed-8c93-02420a0b00f9).

## Abbreviations

VNS: Vagus nerve stimulation
GP: Gaussian processes
FDA: Food and drug administration
TBI: Traumatic brain injury
sVNS: Selective vagus nerve stimulation
eCAP: Evoked compound action potential
eCAPTURS: eCAPs to unravel responses to stimulation
ECG: Electrocardiogram
EMG: Electromyography
HR: Heart rate
BR: Breathing rate
BPM: Beats per minute
DRC: Dose response curve
MAPE: Mean absolute percentage error
EIT: Electrical impedance tomography
IPG: Implantable pulse generator
