# Supplementary material for: Using neural biomarkers to personalize dosing of vagus nerve stimulation
Source: Bioelectron Med. 2024 Jun 17;10:15. doi: 10.1186/s42234-024-00147-4 (PMC11181600; doi:10.1186/s42234-024-00147-4)
Supplement: Supplementary file 1 — Supplementary Material 1. [file 42234_2024_147_MOESM1_ESM.pdf]

Supplementary material for ”Using neural biomarkers to personalize dosing of vagus nerve stimulation”

S1 Physiology

S1.1 Heart rate

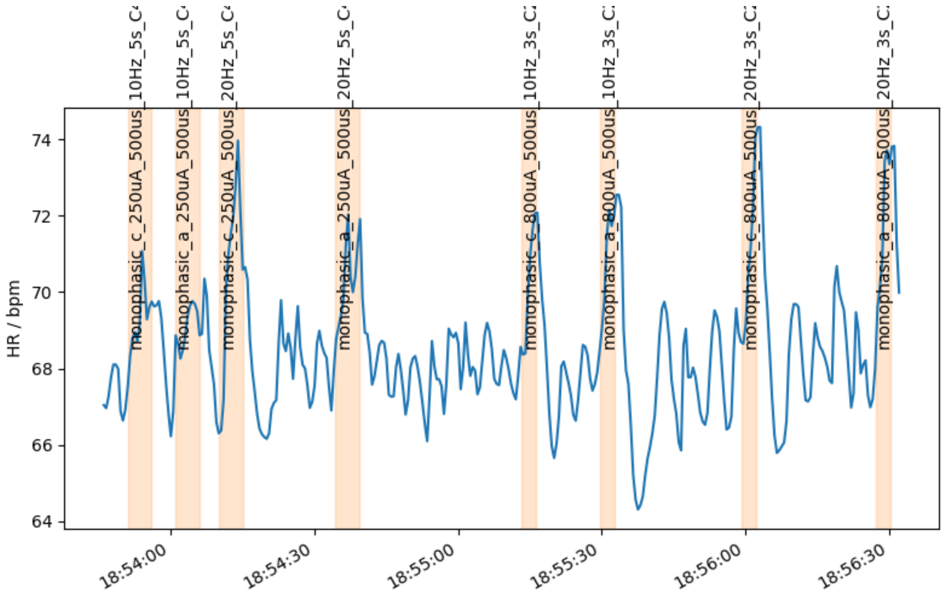

Figure S1: *Example of heart rate responses to VNS for subject S4.*

S1.2 Breathing rate

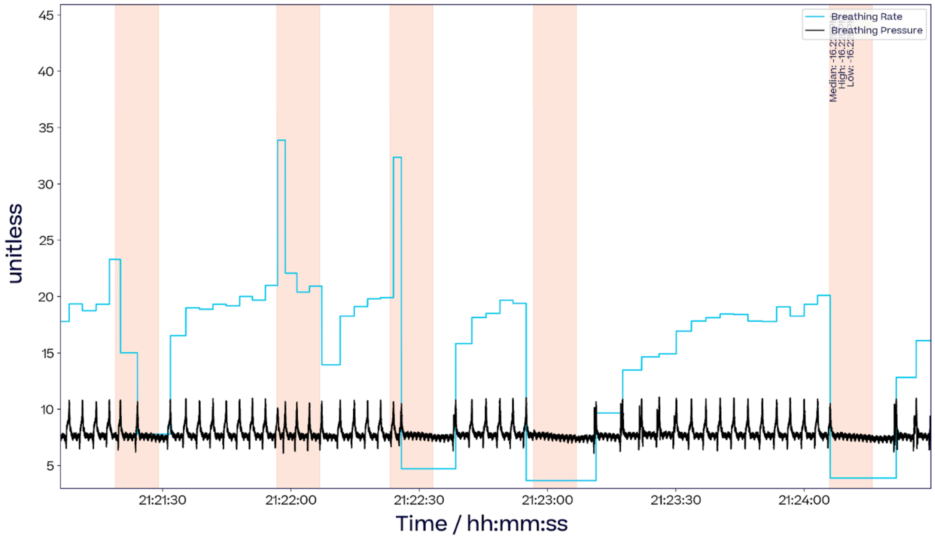

Figure S2: *Example of breathing rate responses to VNS for subject S4.*

S1.3 Activation of Laryngeal Muscles

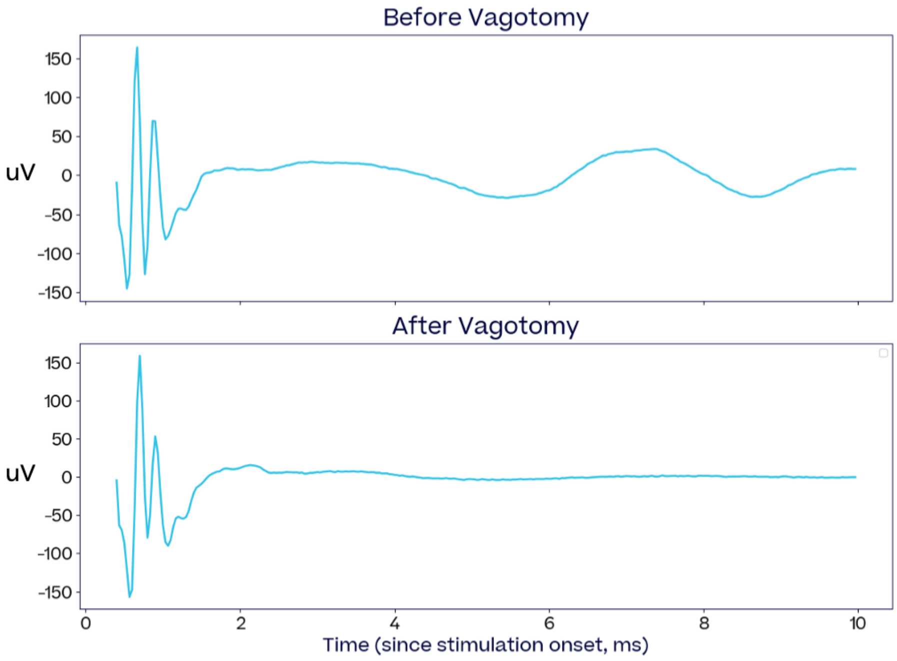

Figure S3: *Effect of caudal vagotomy on laryngeal twitches in neural recordings.* Neural recordings from subject S6 before and after cutting the nerve caudal to the recording and stimulation cuffs. Parameters: 260 µs, 1.0 mA, 10 Hz, 5 s. The muscle artefact occurring at 5-9 ms disappears after caudal vagotomy.

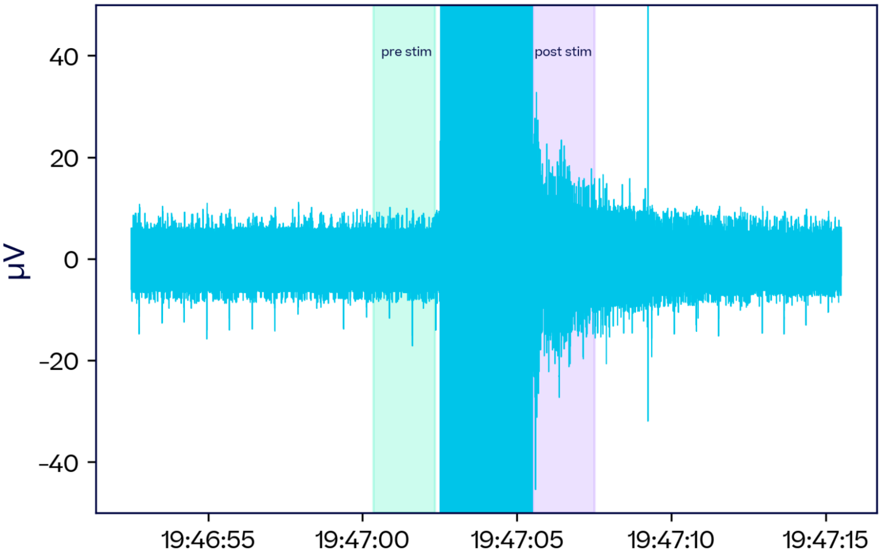

Figure S4: *Illustration of laryngeal spasm.* EMG signal recorded around a stimulation. ΔLEMG is computed as the relative increase in the L_1-norm between the two colored rectangles (1 s before and after the stimulation train).

S2 Neural recordings

S2.1 Recordings of evoked neural activity

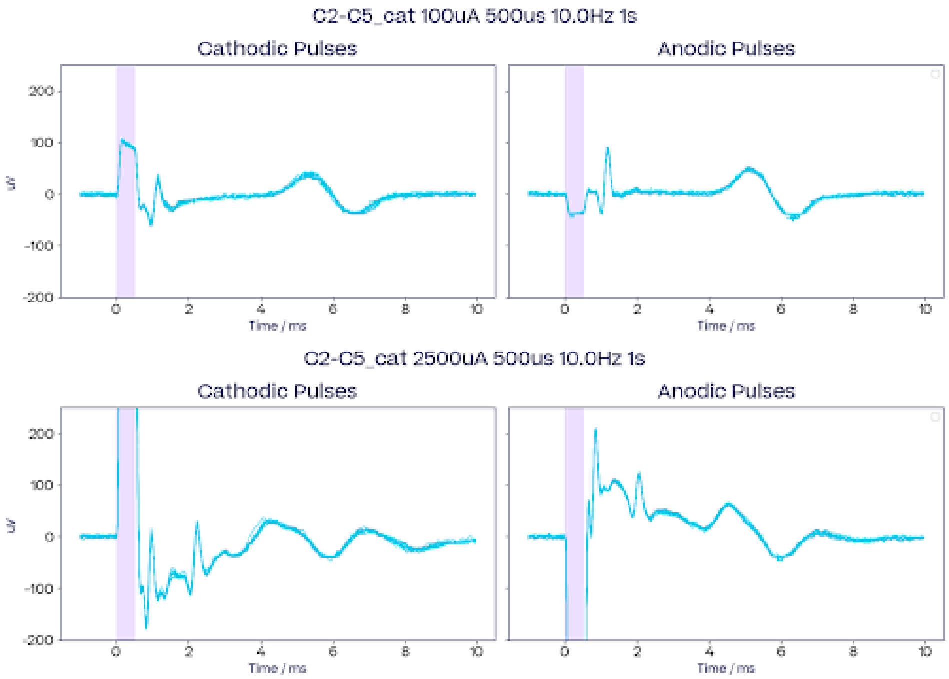

Figure S5: *Raw recordings of* 0.1 mA *and* 2.5 mA *stimulations from S3.* Purple bounds show the duration of the stimulation pulse (500 µs). For high stimulation currents, we observe a slight exponential decrease for the first few milliseconds following the stimulation pulse, due to saturation effects.

S2.2 Comparison of EMG activations from EMG and neural recordings

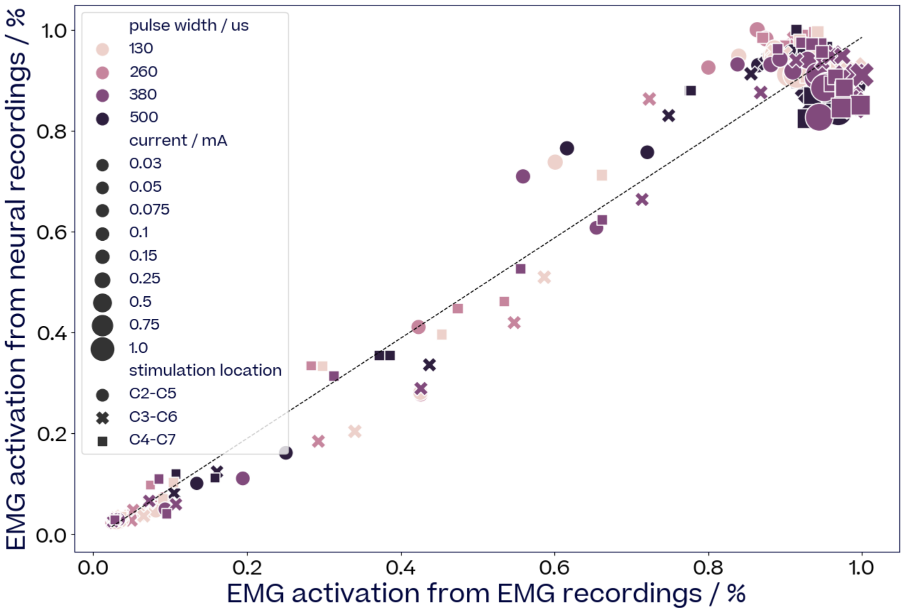

Figure S6: *Comparison of EMG twitches activation computed from EMG and neural recordings in S3.* EMG activations is computed either by taking the L2 norm of the EMG recordings between 2.5 ms and 10 ms after the stimulation pulse (x axis), or by taking the L2 norm of the neurograms between 4 ms and 7.5 ms after the stimulation pulse (y axis). Only stimulations of currents up to 1 mA since B fibre eCAPs can affect the muscle artefact for higher currents. There is a very strong linear relationship between the two EMG activation measurements (black dotted line, R^2=0.67, $p < 1e - 28$).

S2.3 Virtual cathode effect

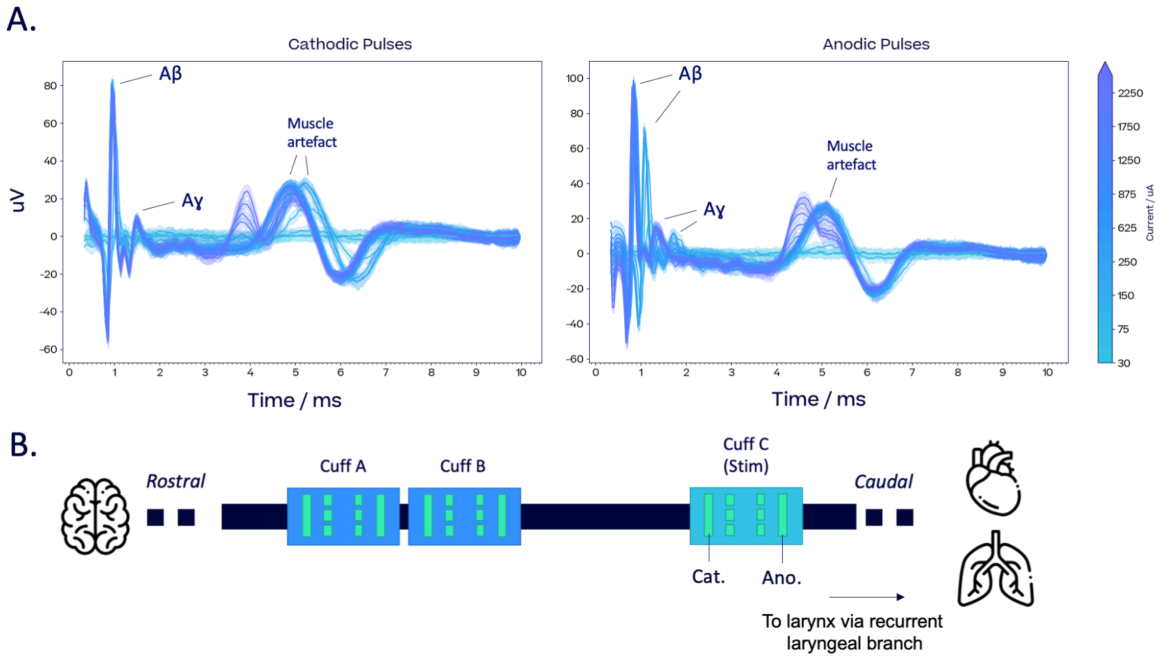

Figure S7: *Illustration of the virtual cathode effect.* Evoked neural activity recorded for increasing currents for subject S3. The virtual cathode effect corresponds to a shift of the depolarisation site in the direction of the anodic site of the stimulation, when current reaches past a certain threshold. This phenomenon can be observed in our recordings in two ways. On the one hand, since our recording cuffs are rostral to the stimulation cuff, this effect appears in our eCAP recordings when the initial depolarisation site (ie, the cathode) is caudal to the anodic site (ie, anodic pulses upper right panel): at around 0.5 mA, Aβ and Aγ eCAPs shift to appear earlier in the recordings, since the depolarisation site of the afferent signal shifts in the rostral direction. On the other hand, the muscle artefact originates from efferent neural activity propagating via the recurrent laryngeal branch. As a result, the shift in location of the depolarisation site can be seen when the cathode is rostral to the anodic site (ie, cathodic pulses, upper left panel).

S3 Custom neural interface

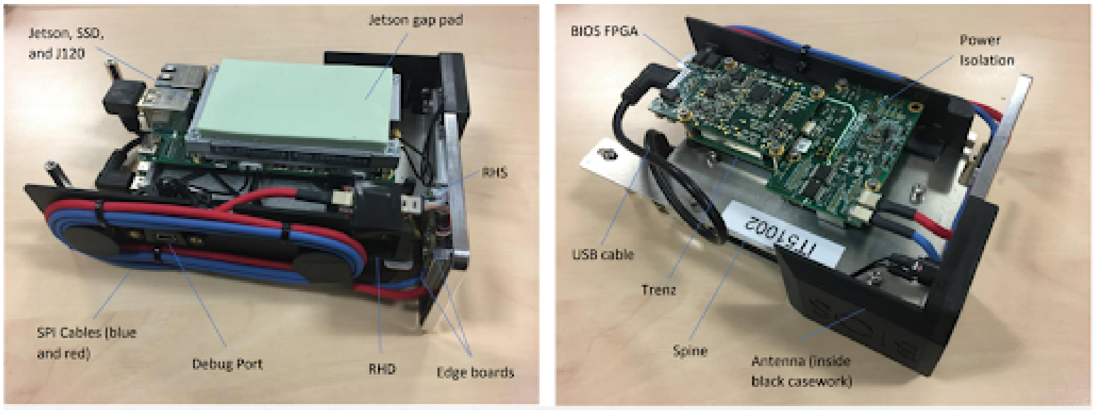

Figure S8: *Interior of BIOS "black box" - customized circuit boards that communicate raw digital signals between a PC-based data acquisition system and the neural interface.*

S4 Stimulation design

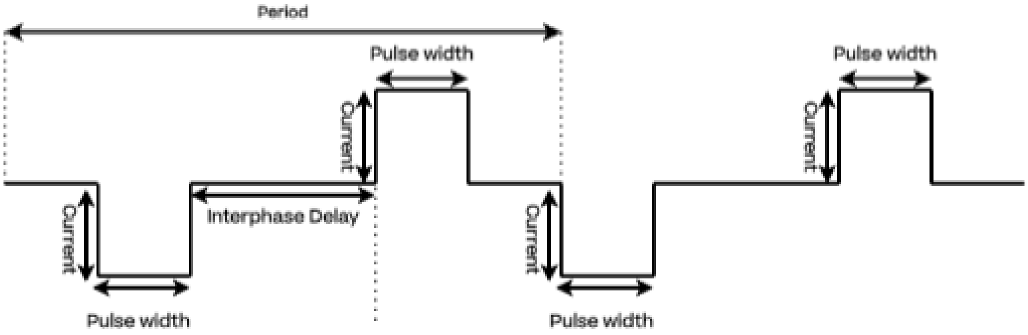

Figure S9: A stimulation consists of a number of identical periods applied immediately one after another. The length of each period is determined by frequency (measured in Hz). The number of stimulation periods is determined by the stimulation "train duration" (measured in seconds) and the frequency. Each period has precisely one or two pulses (where stimulation actually happens). If a period has one pulse, then all pulses in the stimulation have the same polarity and we call the stimulation monophasic. If it has two pulses, then they are identical in every way, except for polarity. In this case we call the stimulation biphasic. Each pulse is rectangular and fully described by the current (measured in mA) and pulse width (measured in microseconds). The time between pulses in a period is determined by interphase delay (measured in microseconds). We always selected interphase delay in such a way that the pulses are all evenly spaced across the duration of the stimulation train.

S5 Inter-subject variability of VNS responses

Fig. S10 compares percentages of changes in heart rate of five subjects in response to VNS for various combinations of currents and frequencies. Subjects show markedly different responses to the same stimulation parameters.

Fig. S11 provides additional comparison between the heart rate response between subjects in response to various combinations of frequencies and train durations.

Importantly, baseline heart rates varied between subjects, which certainly affected responses to these experiments. However, such inter-patient differences in baseline physiological state are inherent to the challenge of inter-subject variability. These examples strongly suggest the need of patient specific VNS parameter adjustments to achieve precise physiological responses.

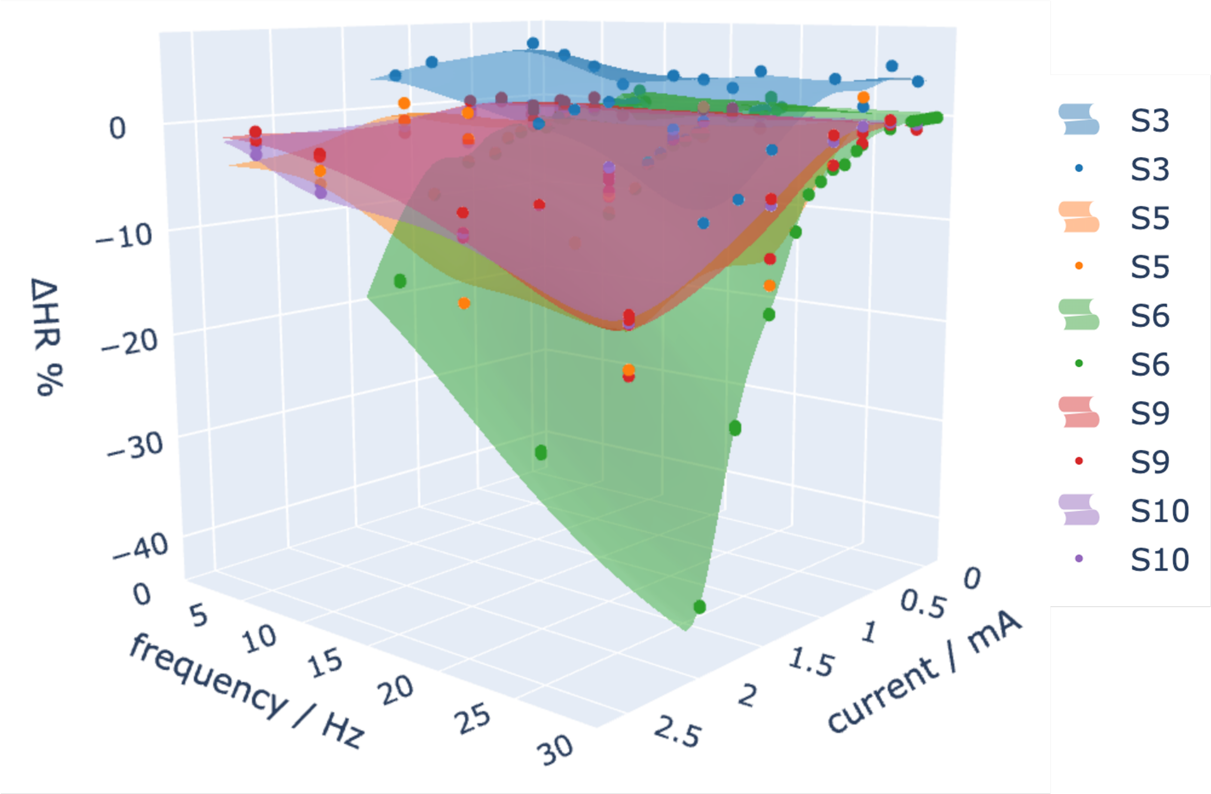

Figure S10: *Comparison of ΔHR responses in five subjects across the (current, frequency) space.* Pulse width was set to either 260us (S7, S10) and 500us (S3, S5, S6); train duration was set to either 1s (S5), 3s (S3) or 5s (S6, S9, S10).

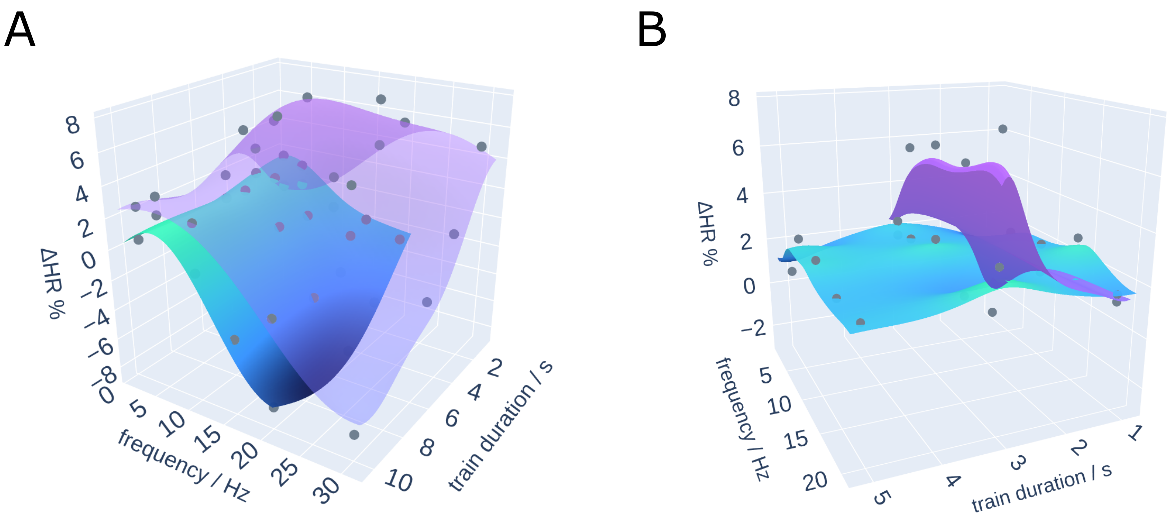

Figure S11: *Comparison of ΔHR response in different subjects.* (A) Subject S3 (purple, baseline HR: 75 bpm) compared to S1 (green, baseline HR: 100 bpm) (current 1.5 mA, pulse width 500 µs), (B) Subject S4 (purple, baseline HR: 65 bpm) to S3 (green, baseline HR: 75 bpm) (current 0.8 mA, pulse width 500 µs,

S6 Limitations of reducing VNS parameters to charge

Stimulation dosage in form of total charge is an important determinant of a therapeutic effect [1, 2]. In Fig. S12b a regression surface (grey) based on total charge is compared to a GP regression surface (green) based on the two separate parameters of frequency and current. This is achieved by computing the charge for each frequency-current input point and retrieving the corresponding response from the regression curve in Fig. S12a. The total charge surface explains most of the variability. One might therefore feel motivated to simplify the search for an optimal VNS dosing by combining several parameters into a single composite metric such as total charge.

However, Fig. S12 also illustrates how such a metric can lead to sub-optimal results. The GP regression surface over the full parameter space has a significantly better fit (F-test, $p < 10^{-}11$) and explains more of the variability of the HR response. If, hypothetically, the aim was to find VNS parameters that maximise tachycardia, the charge curve would suggest an optimum around 3 bpm, while a stimulation based on the expanded current and frequency space would achieve higher tachycardia of around 5 bpm.

This example shows that simplification of the parameter space in order to accelerate parameter searches leads to sub-optimal results compared to a full dimensional search.

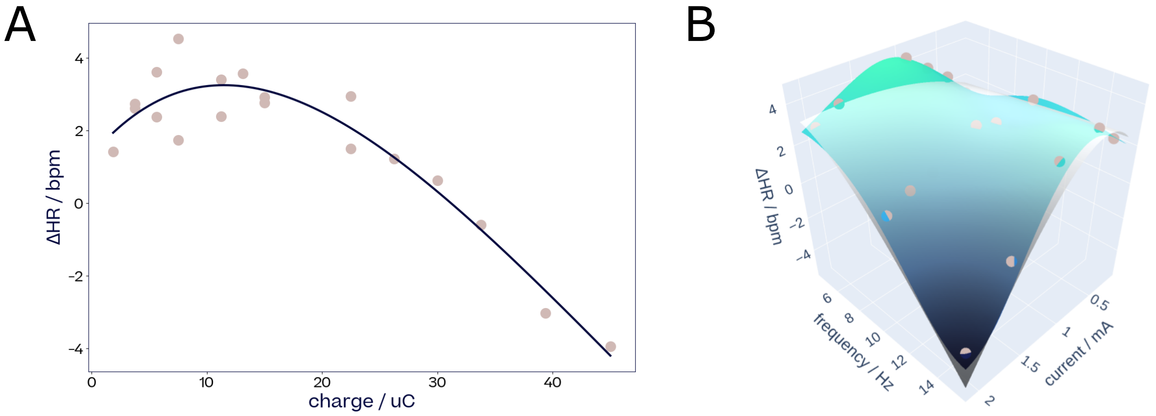

Figure S12: *ΔHR response surface fit based on a single parameter (total charge) compared to fit based on two parameters (current and frequency).* (A) Fit of a two-exponentials function to ΔHR data from subject S3 (train duration 3 s, pulse width 500 µs), this fit is transferred to (B) as grey surface. (B) Two dimensional GP fit (green) to the same data on top of a one dimensional fit (grey) from (A) transferred to two dimensions. The difference in fit is significant (F-test, $p < 10^{-11}$).

S7 Three dimensional exploration of the stimulation parameter space

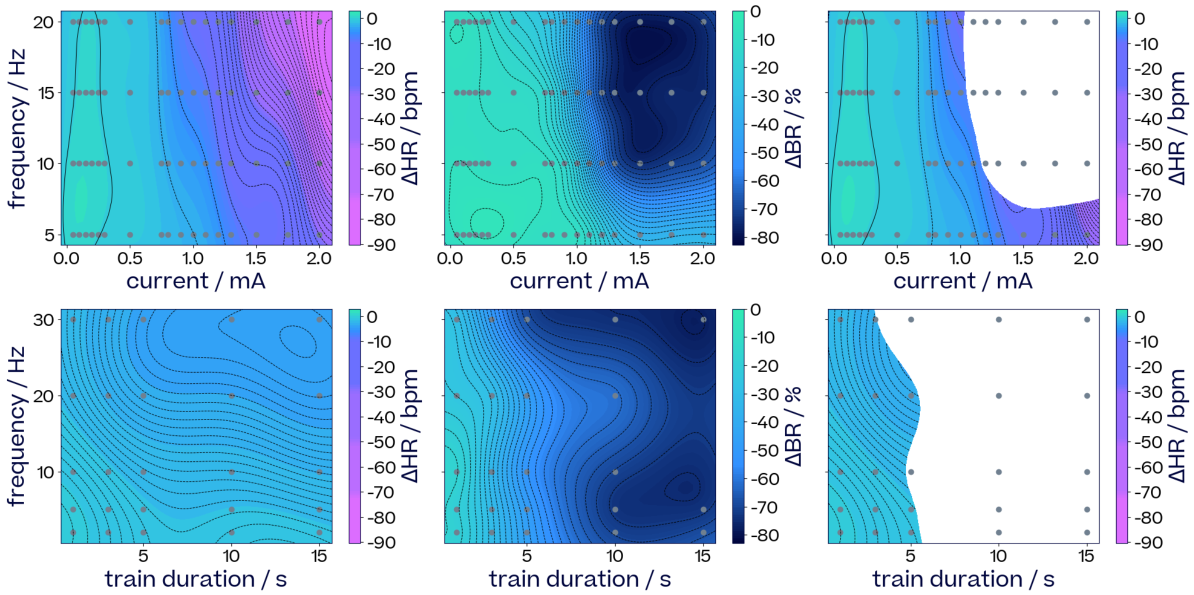

Figure S13: *On-target ΔHR restricted by off-targets, ΔBR across current, frequency and train duration.* Subject S6 ΔHR and ΔBR responses across the frequency-current space (top row, train duration 5 s, pulse width 500 µs), and the frequency-duration space (bottom row, current 1 mA, pulse width 500 µs). The right column shows the space available when ΔBR $> -50\%$.

S8 Invariance of evoked fibre activity across train parameters

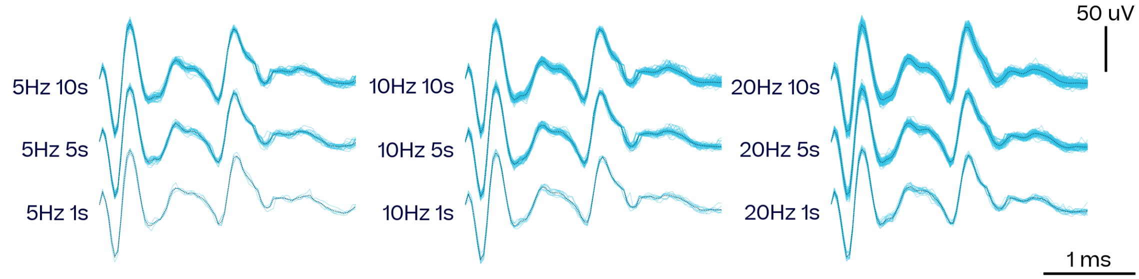

Figure S14: *Invariance of evoked fibre activity with respect to train parameters.* Evoked fibre activity recorded across different frequencies and train durations for fixed current and pulse width (1.5 mA, 500 µs) from subject S3. For each train, both individual pulses (blue lines) and averaged response (black dotted line) are plotted. Mean absolute percentage error (MAPE) of the average response with respect to the 10 Hz, 5 s response: $0.05\% \pm 0.02\%$.

S9 Rheobase transformation of neural thresholds.

Figure S15 shows the neural thresholds presented Figure 6 transformed using the rheobase of Aβ-fibers. For each subject, we approximate the Aβ-fiber rheobase as the median neural threshold for Aβ-fibers observed at 500 µs. We then express the neural thresholds of other fibers and pulse widths as a factor of the Aβ-fiber rheobase.

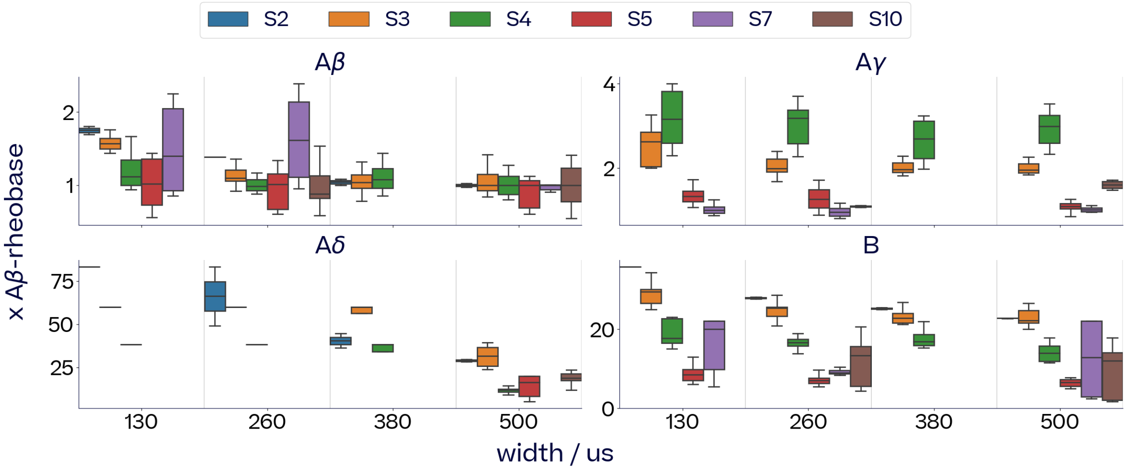

Figure S15: Neural thresholds from Figure 6 expressed as a factor of the Aβ-fiber rheobase.

S10 Evoked fiber activity across pulse parameters

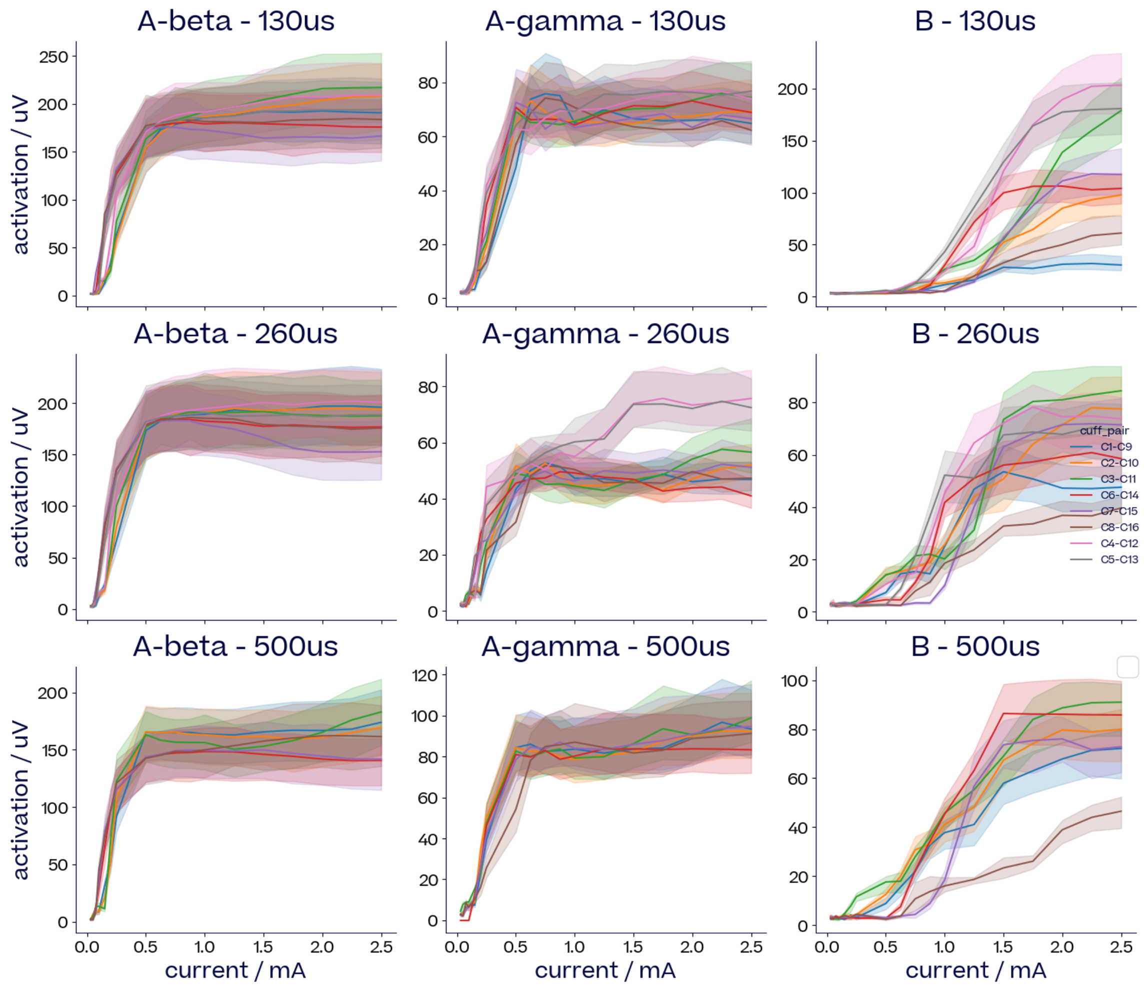

Figure S16: *Dose response curves of Aβ-, Aγ- and B-fibers in Subject S5, across stimulation locations and pulse widths.* Cathodic pulses. Confidence intervals show the 5th and 95th quantiles of each response across the different recording locations.

S11 2D PCA latent space illustration

Figure S17 shows the two-dimensional latent space introduced Figure 7, while zooming in on the region around the longitudinal pairs of subject S5. The different contacts are arranged in loop-like structure.

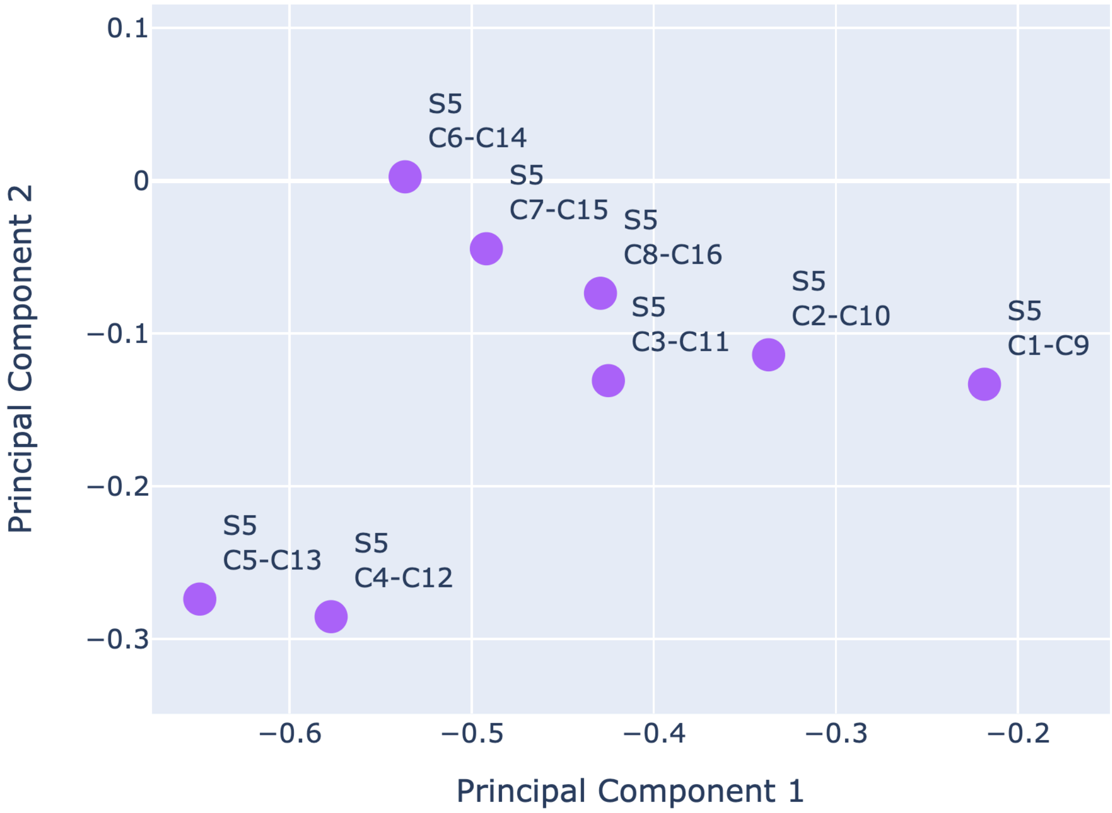

Figure S17: *2D PCA projection of evoked fiber responses as in Figure 7, zoomed in on the region around S5 longitudinal pairs.*

S12 Linearization of HR effect via fiber activations

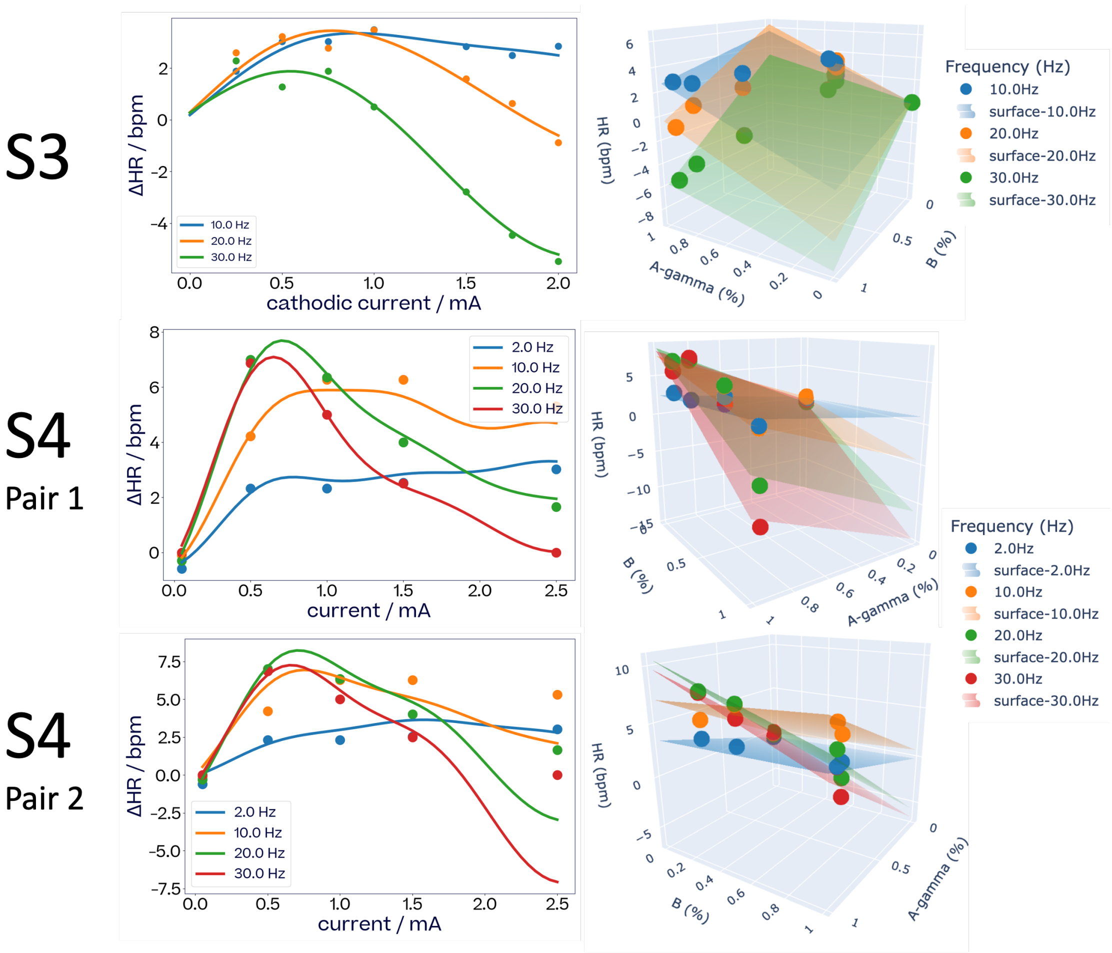

Figure S18: *Left*: Changes in heart rate in response to current and frequency changes of stimuli in S3 and S4 (pulse width 500 µs, train duration 5 s). *Right*: the same change in heart rate, plotted in three dimensions against the activation of Aγ- and B-fibers. 2D linear models are fitted for each frequency. $(0, 0, 0)$ points are added to anchor the surfaces to the origin. All fiber activations are normalised based on the maximum fiber activations recorded across each subject.
